# The structural heterogeneity of AKT autoinhibition

**DOI:** 10.1002/pro.70420

**Published:** 2025-12-23

**Authors:** Liang Xu, Meryem Eren, Jackson Weako, Hyunbum Jang, Ozlem Keskin, Attila Gursoy, Ruth Nussinov

**Affiliations:** ^1^ Computational Structural Biology Section, Frederick National Laboratory for Cancer Research in the Cancer Innovation Laboratory National Cancer Institute Frederick Maryland USA; ^2^ Department of Molecular Biology and Genetics Koç University Istanbul Turkey; ^3^ Computational Science and Engineering Program Koç University Istanbul Turkey; ^4^ Department of Chemical and Biological Engineering Koç University Istanbul Turkey; ^5^ Department of Computer Engineering Koç University Istanbul Turkey; ^6^ Department of Human Molecular Genetics and Biochemistry, Sackler School of Medicine Tel Aviv University Tel Aviv Israel

**Keywords:** AKT, allosteric, autoinhibition, conformational ensemble, conformational heterogeneity, mutation, phosphorylation

## Abstract

AKT is key to controlling cell growth through the PI3K/AKT/mTOR pathway. In the cytosol, in the absence of stimulus, AKT is autoinhibited to prevent uncontrolled activation. Increased AKT activity contributes to tumor growth by phosphorylating numerous downstream targets. Relieving the autoinhibition is a prerequisite for full activation, which occurs through C‐terminal tail phosphorylation by mTOR, followed by activation loop phosphorylation by PDK1. However, the atomic‐level mechanisms by which AKT autoinhibition persists in the cytosol and the phosphorylation (posttranslational modifications) allosterically shift AKT to its open conformation, which may serve as drug targets, remain unclear. Here, we performed explicit molecular dynamics simulations to explore the conformational ensembles of AKT in these different states. Our unbiased results show how the variable loops of the PH domain contribute to the PH‐mediated AKT autoinhibition. Autoinhibited states are commonly only marginally stable, populating function‐related shallow metastable wells with relatively similar energies and low kinetic barriers, making them receptive to regulation. The conformational heterogeneity of AKT's autoinhibitory interface is susceptible to regulation, including by phosphorylation, but also by activating mutations and allosteric inhibitors. As to activation by phosphorylation, allosteric communication between the phosphorylated C‐terminal tail and the PH domain of AKT promotes the release of the PH domain from the kinase domain, independent of PIP_3_. Our results clarify how mutations and phosphorylation can impact autoinhibition and resistance to allosteric inhibitors, highlighting how metastable states can contribute to cellular regulation. Heterogeneous population with low stability and low kinetic barriers can be a useful attribute of living cells.

## 1 INTRODUCTION

The serine/threonine protein kinase AKT (also known as protein kinase B or PKB) is pivotal in the PI3K/AKT/mTOR signaling pathway (Manning & Cantley, 2007; Manning & Toker, 2017; Nussinov et al., 2025; Vivanco & Sawyers, 2002). AKT is a member of the AGC (protein kinases A, G, and C) superfamily, consisting of three distinct but highly homologous isoforms: AKT1, AKT2, and AKT3 (Gonzalez & McGraw, 2009; Hanada et al., 2004). AKT1 (below referred to as AKT) is ubiquitously expressed in tissues and together with mTORC1, which helps activate, promotes ribosomal RNA and protein synthesis, making it a major factor in cell growth, proliferation, and survival. Through phosphorylation of over 100 substrates, it modulates multiple cellular processes (Hanada et al., 2004; Manning & Toker, 2017; Nussinov et al., 2025). Aberrant AKT activation leads to diseases, including cancer and type‐2 diabetes (Gonzalez & McGraw, 2009; Hassan et al., 2024; He et al., 2021).

**FIGURE 1:**
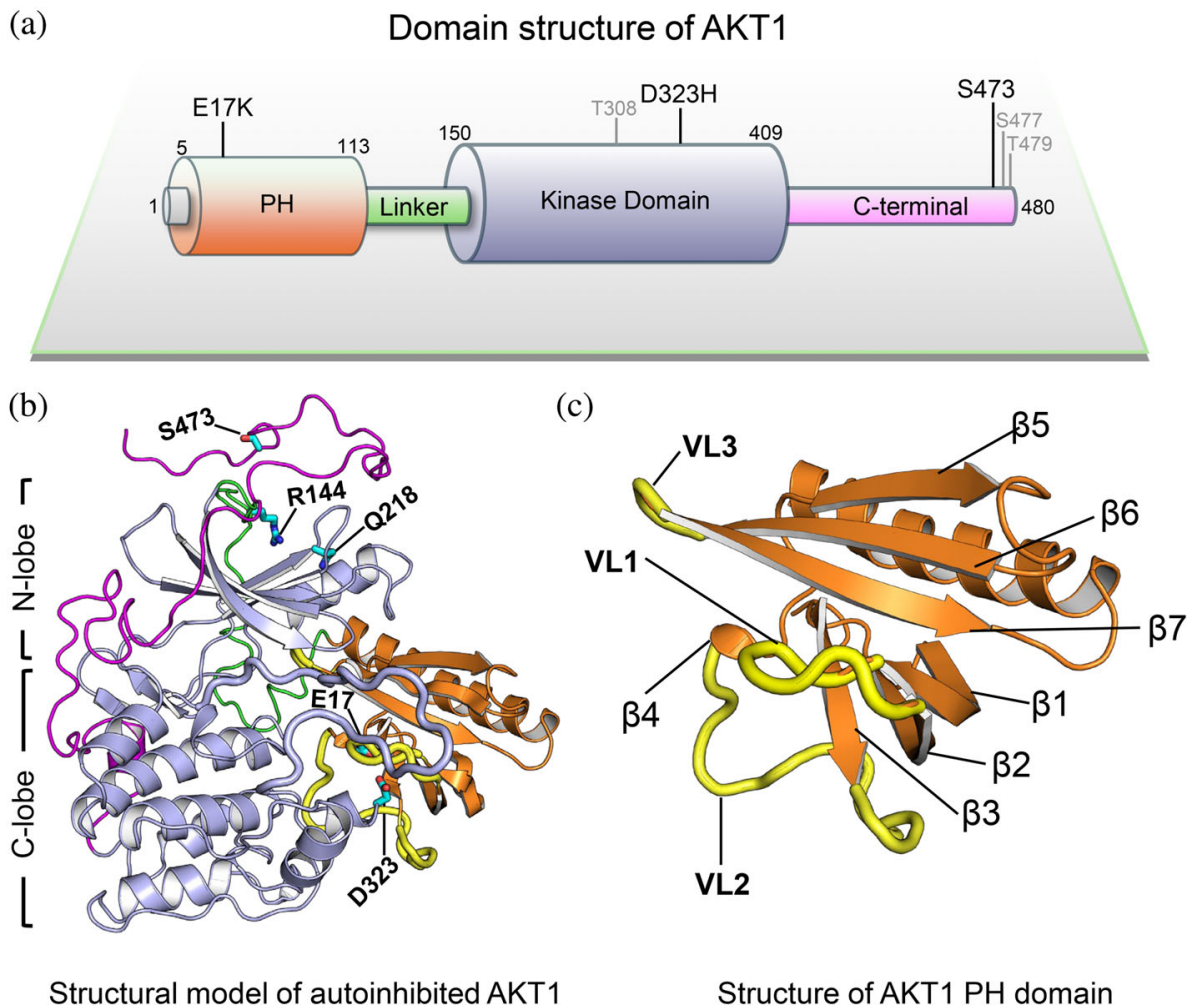
Structure of AKT. (a) Domain structure of AKT. In the domain structure, the phosphorylation site at T308, S473, S477, and T479, and the two mutations, E17K and D323H, are marked. Phosphorylation of T308, S477, and T479 are not studied in this work. (b) The model structure of the full‐length AKT in autoinhibition, based on the crystal structure of AKT bound with an allosteric inhibitor (PBD ID: 6S9W). The key residues, E17 in the PH domain (orange), Q218 and D323 in the kinase domain (blue), R144 in the linker region (green), and S473 in the C‐terminal tail (purple) are labeled. The activation loop of the kinase domain is highlighted by blue tube representation. The three variable loops are highlighted by yellow tube representation. The N‐lobe and C‐lobe of the kinase domain are characterized by containing multiple β‐strand and α‐helical structures, respectively. (c) The initial conformations of AKT PH domain, including the variable loop 1 (VL1, residues 15–22) between β1 and β2, the variable loop 2 (VL2, residues 38–53) between β3 and β4, and the variable loop 3 (residues 79–82) between β6 and β7. Note that β4 becomes unstable in the full‐length autoinhibited AKT.

In addition to a conserved kinase domain, AKT consists of an N‐terminal pleckstrin homology (PH) domain that is responsible for the recruitment of AKT to the plasma membrane (Figure 1a) (Bellacosa et al., 1998). AKT is activated by phosphorylation on its activation loop (T308) and C‐terminal hydrophobic motif (S473) by PDK1 and mTORC2, respectively (Sarbassov et al., 2005; Stokoe et al., 1997). AKT activation can also take place through phosphorylation at its extreme C‐terminal tail (S477 and T479) by cyclin‐A/CDK2 (Liu, Begley, et al., 2014; Liu, Wang, & Wei, 2014). The interaction of its PH and kinase domains triggers and sustains its inactive autoinhibited state in the cytosol (Calleja et al., 2007). Stabilization of AKT in the autoinhibited conformation has motivated the development of allosteric inhibitors (Pervanidis et al., 2024) (Figure S1). Recently, covalent inhibitors that selectively target the most common oncogenic AKT mutant E17K were reported (Craven et al., 2025). The crystal structure of autoinhibited AKT stabilized by a nanobody resembles the structure of full‐length AKT predicted by AlphaFold2 (Jumper et al., 2021; Varadi et al., 2022), and both show a rather weak interaction between the kinase and PH domains (Truebestein et al., 2021). However, notably, this crystal structure biases the conformational ensemble of free AKT as it was crystallized with an altered linker between the kinase and PH domains (Truebestein et al., 2021), leaving the detailed conformational state of native AKT still unresolved. Moreover, PDK1, like AKT, possesses a PH domain, but its autoinhibited states have not been captured experimentally (Leroux & Biondi, 2023), raising the vital question of how the structurally similar AKT and PDK1, two key protein kinases with tens of substrates, modulate the PH domain‐mediated autoinhibition.

Phosphorylation of the activation loop at T308 drives AKT to adopt an active conformation essential for phosphoryl transfer from ATP to the substrate (Clark & Cullati, 2025). While no specific order of events, phosphorylation of the C‐terminal S473 likely tends to precede the phosphorylation of the activation loop of AKT. It stabilizes the active conformation of AKT by leading to the interaction of the C‐terminal tail with the N‐lobe of the kinase domain (Calleja et al., 2007; Calleja et al., 2009; Cole et al., 2019; Lin et al., 2012; Yang et al., 2002). Protein semisynthesis experiments using expressed protein ligation further indicated that phosphorylation of S473 (pS473) can relieve autoinhibition and activate AKT by interacting with R144 in the PH‐kinase domain's linker and Q218 in the N‐lobe of the kinase domain (Figure 1b; Chu et al., 2018). Such allosteric communication between the C‐terminal tail and the N‐terminal PH domain is lipid‐independent, whereas other experimental evidence suggests that the binding of signaling phospholipid PIP_3_ to AKT leads to the displacement of the PH domain from its autoinhibited conformation, activating AKT (Lucic et al., 2018; Siess & Leonard, 2019). In the presence of an allosteric inhibitor, the PH domain can bury 1526 Å^2^ surface area between the PH and kinase domains (Wu et al., 2010), and the interaction between pS473 and R144 seems unable to disrupt this interface. In the absence of inhibitors, the interaction between the domains is expected to be less tight. We expected that exploring the unbiased driving force at the autoinhibitory interface of AKT will allow us to elucidate the conformational mechanism of how the C‐terminal phosphorylation relieves its autoinhibition.

The PH domain of AKT comprises three variable loops, involving residues 15–22 (variable loop 1, VL1), 38–53 (variable loop 2, VL2), and 79–82 (variable loop 3, VL3) (Figure 1c). AKT mutations in the PH domain including E17K, D32Y, D46E, P51L, L52R/F, Q79K, and W80R predominantly occur in these unstructured regions (Yi & Lauring, 2016). Several activating mutations such as E17K, L52R, Q79K, and D323H can disrupt the autoinhibitory interface of AKT and confer resistance to allosteric AKT inhibitors (Manning & Cantley, 2007; Parikh et al., 2012). The mutations were observed to differentially affect AKT activation (Ebner et al., 2017). AKT with the E17K mutation (AKT^E17K^) is largely maintained in the autoinhibited state, which can be trapped and stabilized by covalent allosteric inhibitors (Craven et al., 2025). Although the PH domain of AKT^E17K^ binds PIP_2_ approximately 25 times more weakly than PIP_3_, the increased activity of AKT^E17K^ is partially attributed to the enhanced affinity of the PH domain for PIP_2_ compared to the wild type (Bae et al., 2022). In contrast, D323, which is close to the activation loop of the kinase domain, seems more involved in sustaining the autoinhibited state of AKT. The double AKT mutant D323A/D325A most likely disengages the PH domain from the kinase domain (Ebner et al., 2017). Analysis of available AKT crystal structures with allosteric inhibitors shows that D323 could establish electrostatic interactions with K14, R23, or R25 on or in the vicinity of VL1 in the PH domain (Figure S2 and Table S1). It is thus interesting to see how these variable loops modulate the interactions of the PH domain with the kinase domain of AKT in response to diverse pathological and engineered mutations, and whether these could inform allosteric drug design.

Here, guided by experimental data, we explored the conformational ensembles of full‐length AKT in its autoinhibited state by performing all‐atom molecular dynamics (MD) simulations. Our unbiased simulation results show that the variable loops of the PH domain predominantly contribute to the association of the PH domain with the kinase domain. Mutant residue K17 in AKT^E17K^ can establish new interactions with the activation loop of the kinase domain, sustaining the autoinhibition. In contrast, AKT with the D323H mutation (AKT^D323H^) severely disrupts the autoinhibitory interface. Notably, in the structure of AKT with pS473 (AKT^pS473^), we identified the allosteric communication pathway between the C‐terminal tail and the kinase domain triggered by the interaction of pS473 with R144, which can release the autoinhibition, an occurrence not observed in the wild‐type AKT (AKT^WT^). Collectively, our results suggest the essential role of conformational heterogeneity in regulating AKT and provide mechanistic insights into the distinct functional consequences of AKT mutations. Our results suggest that conformational heterogeneity is a key property of autoinhibition scenarios. Heterogeneity implies function‐related metastable states teetering stability with low kinetic barriers, making it sensitive to disruption in the living cell.

## 2 RESULTS

We performed MD simulations on full‐length AKT in an autoinhibited state to investigate how C‐terminal tail phosphorylation (pS473), PH domain mutation (E17K), and kinase domain mutation (E323H) affect it. We monitored the effects of these posttranslational modifications at the atomic level by comparing them to the wild‐type AKT in an autoinhibited state. AKT activity requires phosphorylation of the activation loop at T308. The kinase–PH domain interaction in the autoinhibited AKT prevents PDK1 from effectively accessing and phosphorylating T308 (Shaw & Burke, 2025). Therefore, we did not include phosphorylated T308 in our models. Three replicate simulations (each 1 μs) were carried out for each system. Below, we detailed the analyses and results.

### 2.1 Phosphorylation and mutations lead to distinct conformational dynamics of AKT

**FIGURE 2:**
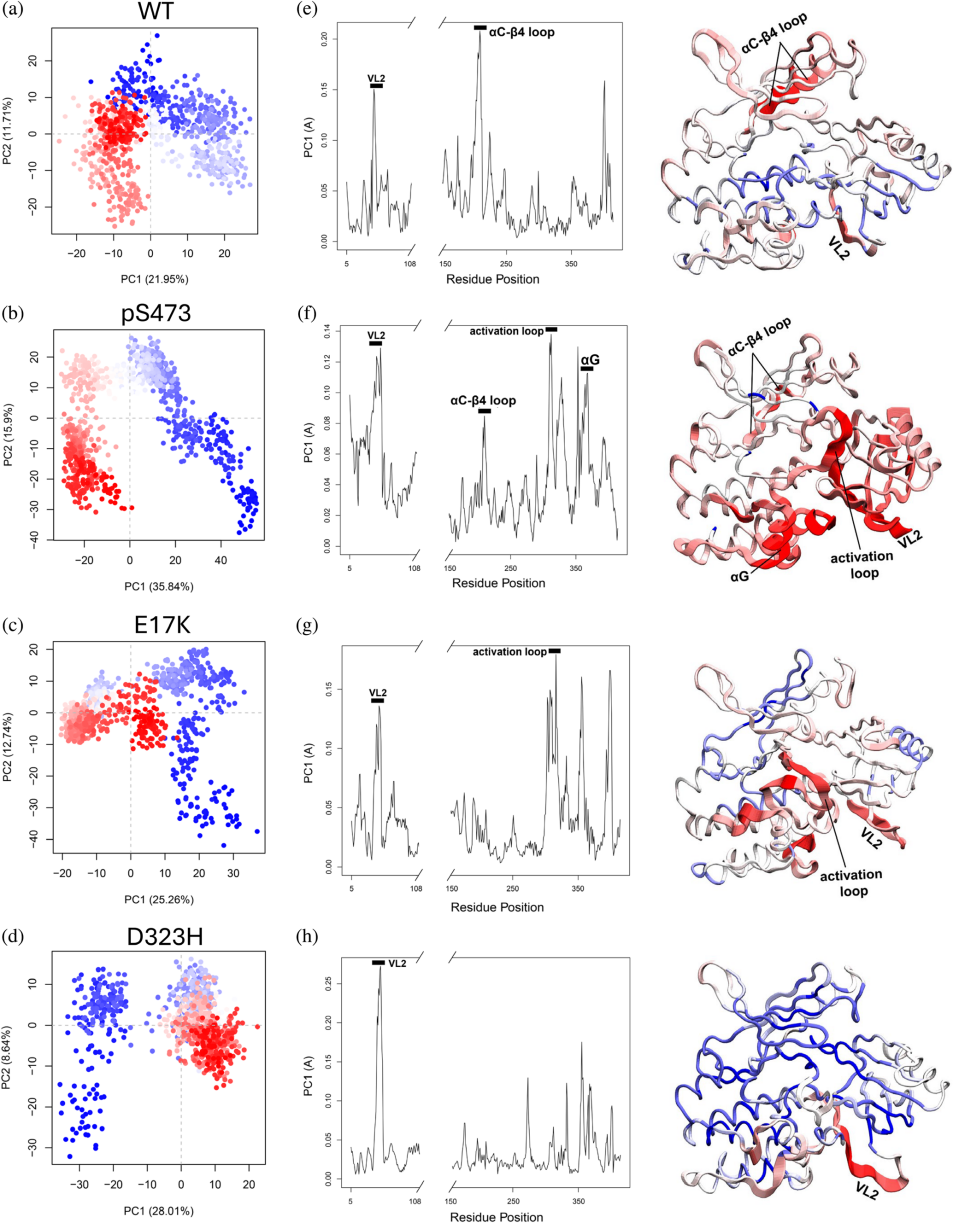
C‐terminal phosphorylation (pS473) and mutations (E17K and D323H) sufficiently bias the conformational ensembles of AKT. Principal component analysis (PCA) for (a) AKT^WT^, (b) AKT^pS473^, (c) AKT^E17K^, and (d) AKT^D323H^. The percentage of the first two components (PC1 and PC2) accounting for the total variance is shown in parentheses. Each dot represents one snapshot of the trajectory and the color from blue to red represents the trajectory frame from beginning to end. PC1 loading plots for (e) AKT^WT^, (f) AKT^pS473^, (g) AKT^E17K^, and (h) AKT^D323H^. Regions with high conformational variability have more contribution to the PC1. The linker and C‐terminal regions are disordered and excluded for PCA. Tube representations of the PC1 from PCA are shown for each system, with the color gradients from immobile (blue) to highly mobile (red). The αG‐helix in AKT^pS473^ significantly contributes to PC1, indicating the large dynamics of the C‐lobe of the kinase domain.

We first performed principal component analysis (PCA) on the whole trajectory of each system to monitor the changes in the conformational dynamics over the simulations. Each trajectory was aligned to the starting conformation in terms of the coordinates of the Cα atoms of the kinase and PH domains (the disordered C‐terminal and linker regions were excluded). The positions of Cα atoms were then transformed into a set of principal components that capture the directions of the largest variance in the conformational ensemble and display the most significant motions. We observed a clear separation of the conformations over time for AKT^WT^ (Figure 2a), suggesting a large deviation from the initial conformation. The PCA analysis shown in Figure 2 is based on a single trajectory, while similar results from the other two replicate simulations are in Figures S3–S6. The substantial conformational deviation from the starting models was also demonstrated by the fluctuations in root‐mean‐square deviation (RMSD, Figures S7–S10). As the starting structure was stabilized with an allosteric inhibitor, this result highlights that the binding of allosteric inhibitors can significantly bias the conformations of AKT. Compared to AKT^WT^, the distinct distributions of conformations sampled by AKT^pS473^ (Figure 2b), as well as E17K (Figure 2c) and D323H (Figure 2d), indicate that C‐terminal phosphorylation and single‐point mutations bias the conformational ensemble. Because the first principal component (PC) captures the highest percentage of the total mean square displacement of Cα atom positional fluctuations (David & Jacobs, 2014), we examined the contribution of each residue (excluding the unstructured C‐terminal and linker regions) to the first PC (Figure 2e–h). We found that the VL2 of the PH domain contributes to the conformational fluctuations in all systems, suggesting that this variable loop largely retains a disordered structure at the autoinhibitory interface. The formation of a short *α*‐helix in VL2 (residues 44–49) was observed when the head group of PIP_3_ was bound to the isolated PH domain (Milburn et al., 2003). However, such a conformational change was not observed in the present autoinhibited AKT. In addition to the VL2, we also identified the αC‐β4 loop that contributes to the structural fluctuations in AKT^WT^ (Figure 2e) and AKT^pS473^ (Figure 2f), but not in AKT^E17K^ (Figure 2g) and AKT^D323H^ (Figure 2h). The αC‐β4 loop is a conserved structural motif in kinase; a recent work of PKA suggested that the αC‐β4 loop is critical for ATP binding, and the opening and closing of the catalytic cleft of the kinase domain (Wu et al., 2024). Here, the distinct dynamics of the αC‐β4 loop imply that C‐terminal phosphorylation and AKT mutants can regulate the kinase via different mechanisms. Of interest, the activation loop (residues 292–319) undergoes a larger conformational change in AKT^pS473^ and AKT^E17K^, thus contributing to the variance of positional fluctuations along the first PC. For AKT^pS473^, we also observed that the αG‐helix (residues 353–364), which is in the C‐lobe of the kinase domain, displays more pronounced dynamics, compared to other systems.

**FIGURE 3:**
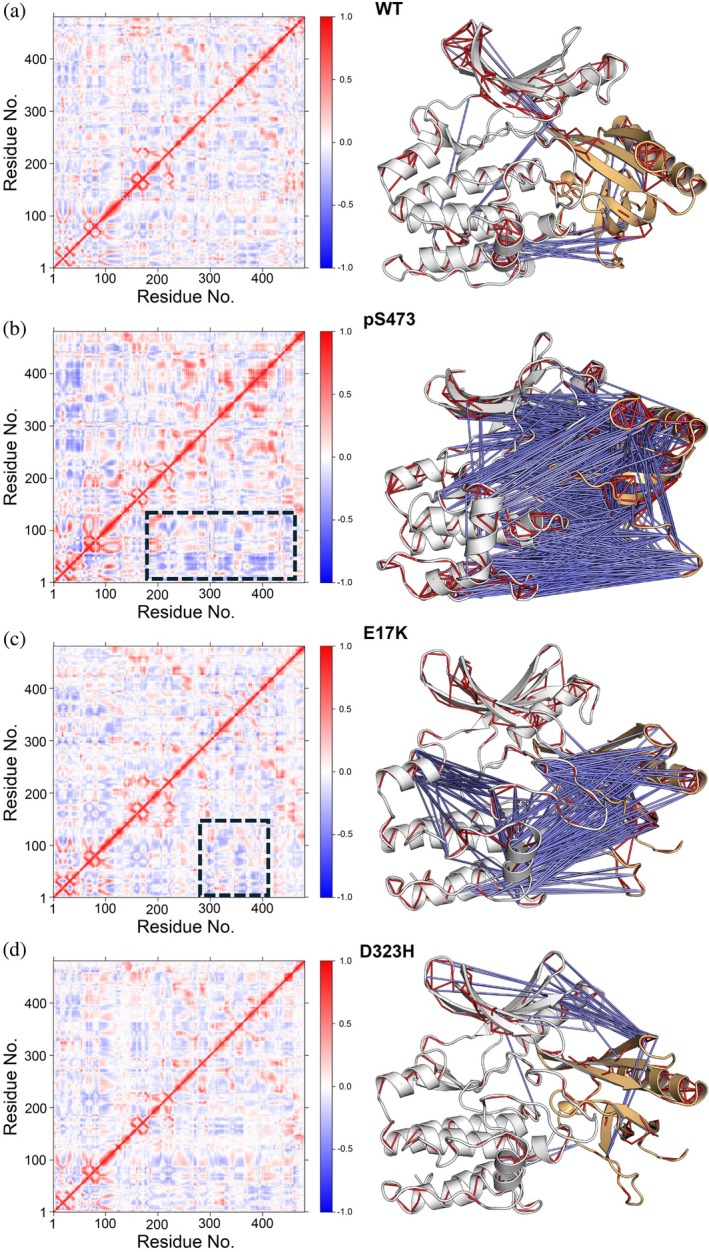
C‐terminal phosphorylation (pS473) and mutations (E17K and D323H) lead to distinct correlated motions between the PH and kinase domains. Dynamic cross‐correlation map (DCCM) calculated for (a) AKT^WT^, (b) AKT^pS473^, (c) AKT^E17K^, and (d) AKT^D323H^. The PH domains of AKT^pS473^ and AKT^E17K^ with significant changes in the anticorrelated motions are highlighted with a rectangle. The Positively correlated motions are colored red; and the negatively correlated motions are colored blue. The conformational representations show the most correlated motion with the *C*_*ij*_ in [0.8, 1.0], and the anticorrelated motion with the *C*_*ij*_ in [−0.6, −0.4]. The most anticorrelated motion with the *C*_*ij*_ in [−0.8, −0.6] is only identified in AKT^pS473^ and shown in Fig. S11. The significant anticorrelation between the PH and kinase domains in AKT^pS473^ suggests the opposite movement of the PH domain with respect to the C‐lobe of the kinase domain. For each system, the analysis is based on the last half trajectories of three replicas.

The above PCA quantifies the most important large‐scale motions in the conformational dynamics of the autoinhibited AKT. To further characterize the correlation of the dynamics of different regions within AKT, we calculated the pairwise cross‐correlation coefficients *C*_*ij*_ to obtain the dynamic cross‐correlation matrix (DCCM) for each system (Figure 3). The most anticorrelated motion with a *C*_*ij*_ between −0.8 and −0.6 only occurred in AKT^pS473^ (Figure S11). For a better comparison, we mapped the positively correlated motions with a *C*_*ij*_ between 0.8 and 1 and the negatively correlated motion with a *C*_*ij*_ between −0.6 and −0.4 to the structure of AKT. Since we are interested in the effects of phosphorylation and mutations on the conformational changes of the autoinhibited state, we focus on the correlation between the kinase and PH domains. Compared to AKT^WT^ (Figure 3a), AKT^pS473^ demonstrates the most significant negative correlations between the PH and kinase domains (Figure 3b). In particular, the anticorrelated motions between the VL2 of the PH domain and the C‐lobe of the kinase domain indicate that the two regions move in opposite directions over the simulations, tending to displace the PH domain from the kinase domain. However, these negatively correlated motions become weaker in AKT^E17K^ (Figure 3c) and vanish in AKT^D323H^ (Figure 3d). We also observed the anticorrelated motion between the PH domain and the activation loop of the kinase domain in AKT^E17K^, suggesting that coupling of the PH domain with the activation loop may destabilize the autoinhibited state. In addition, we identified a relatively weak anticorrelated motion between the PH domain and the N‐lobe of the kinase domain in AKT^D323H^, which may promote the dissociation of the PH domain from the kinase domain differently from AKT^E17K^. Taken together, our conformational dynamic analyses reveal that the binding of allosteric inhibitors significantly biases the autoinhibited conformational ensemble. The fluctuations of the VL2 predominantly contribute to the dynamics of the PH domain in all systems, whereas the extent to which the αC‐β4 loop, the activation loop, and the αG‐helix contribute to the essential dynamics of the kinase domain varies depending on the structural alterations. The notably anticorrelated motions between the PH and kinase domains of AKT^pS473^ tend to attenuate their association.

### 2.2 The variable loops of the PH domain contribute to the heterogeneity of the autoinhibitory interface

**FIGURE 4:**
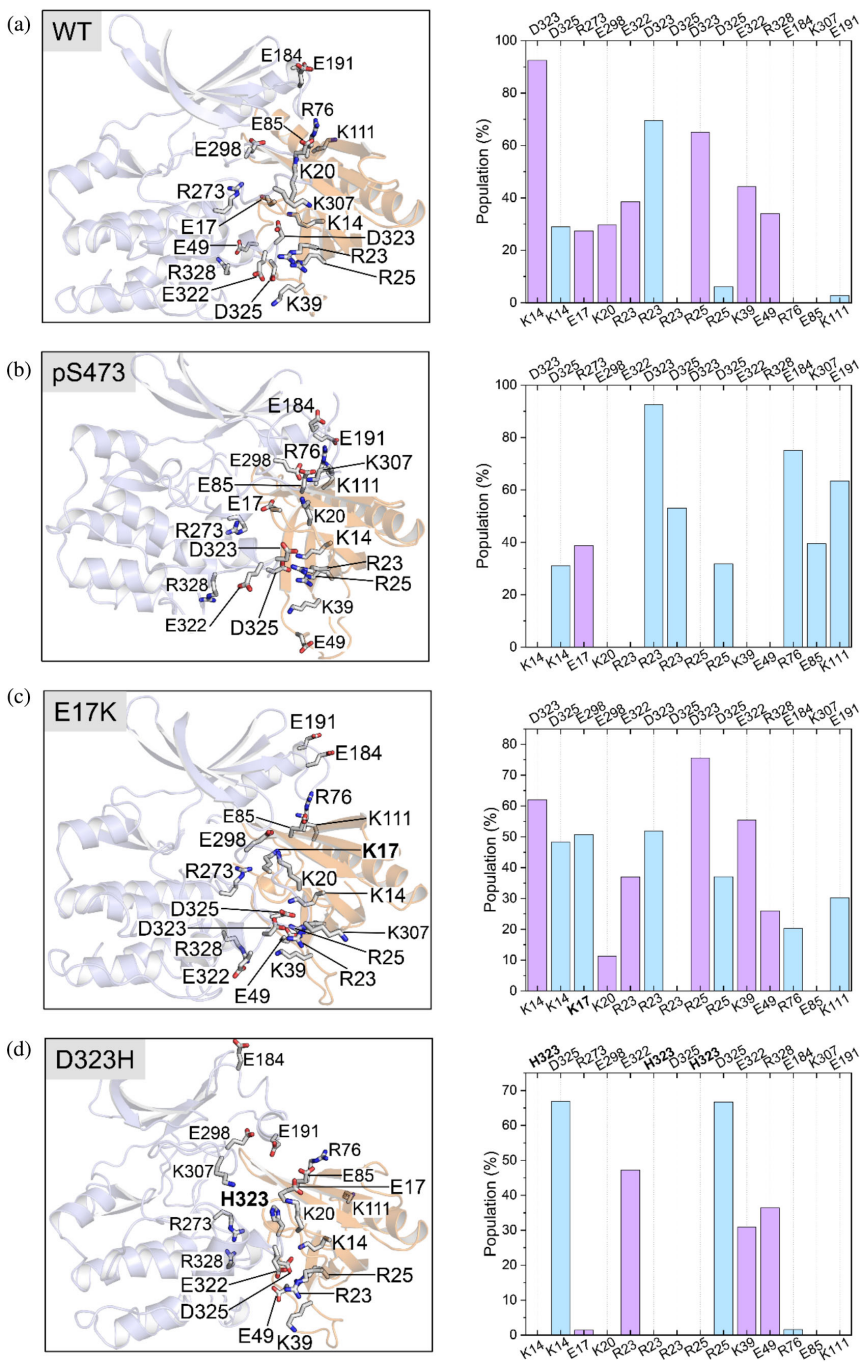
C‐terminal phosphorylation pS473 and mutations E17K and D323H differently alter the autoinhibitory interface. The strength of electrostatic interactions at the autoinhibitory interface are characterized by the populations of the salt bridges for (a) AKT^WT^, (b) AKT^pS473^, (c) AKT^E17K^, and (d) AKT^D323H^. The kinase and PH domains are colored blue and orange, respectively. The magenta bar indicates that these salt bridges are present in the starting structure, and the light blue bar indicates that these salt bridges are newly formed during simulations. Compared to AKT^WT^, multiple salt bridges between the C‐lobe of the kinase domain and PH domain disappeared, whereas new interactions (R76–E184, K111–E191, and E85–K307) emerged between the N‐lobe of the kinase domain and PH domain, implying that pS473–R144 interaction strengthens the interaction of the PH domain with the N‐lobe of the kinase domain, but weakens the interaction of the PH domain with the C‐lobe of the kinase domain. For each system, the analysis is based on the last half trajectories of three replicas.

To examine how distinct conformational dynamics impact the association of the PH and kinase domains, we characterized the electrostatic interactions at the autoinhibitory interface by calculating the populations of relevant salt bridges (Figure 4). In the initial structure of AKT bound to the allosteric inhibitor (PDB: 6S9W, Figure S2 and Table S1), K14, E17, K20, R23, and R25, which are on or in the vicinity of VL1 in the PH domain, establish electrostatic interactions with D323, R273, E298, E322^main‐chain^, and D323^main‐chain^ of the kinase domain, respectively. In addition, K39 and E49 of VL2 in the PH domain also interact with E322 and R328 of the kinase domain, respectively. However, after the structure relaxes in the absence of the inhibitor, the salt bridge interactions of E17–R273, K20–E298, and E49–R328 are less populated (<50%) in AKT^WT^ (Figure 4a), and the K39–E322 salt bridge becomes weaker (~51%). Since K39 and E49 are in VL2, the reduced interactions with the kinase domain result in decreased stability of VL2, as demonstrated by its large fluctuations in PCA (Figure 2a). In contrast, the association of VL1 with E322 and D323 of the kinase domain is relatively stable, contributing to the autoinhibited conformation. In AKT^pS473^, however, R23 predominantly contributes to the association of VL1 with the kinase domain (Figure 4b). Compared to AKT^WT^, the loss of stable interactions of K14–D323, R25–E322/D323, and K39–E322 significantly weakens the association of VL1/VL2 with the kinase domain. This is also evident in the anticorrelated motion between the variable loops and the C‐lobe of the kinase domain (Figure 3b). Formation of new interactions between R76 and E184, and between E85 and K307 appears to strengthen the association of VL3 with the kinase domain, as R76 and E85 are located near this short loop (residues 79–82) (Figure 4b). Additionally, the K111–E191 interaction further drives the PH domain closer to the N‐lobe of the kinase domain. The pattern of salt bridge interactions in AKT^E17K^ resembles that of AKT^WT^ (Figure 4c), but K14 switches to interact with D325 rather than D323 in AKT^WT^. The mutant residue K17 forms a relatively stable salt bridge with E298, which is maintained in the presence of an allosteric inhibitor (Figure S2) (Craven et al., 2025). The salt bridges formed between the same residues in the variable loops and the kinase domain of AKT^E17K^ are generally less populated than those in AKT^WT^, indicating that the autoinhibitory interface is less stable in AKT^E17K^. A more unstable association between the PH and kinase domains was observed in AKT^D323H^ (Figure 4d). Replacing D323 with a histidine also disrupts its neighboring interactions of E322 with the variable loops of the PH domain. The remaining interactions include K14–D325, and R25–D325, with D325 being sandwiched between K14 and R25. The electrostatic association between VL2 and the kinase domain (K39–E322 and E49–R328) in AKT^WT^ and AKT^E17K^ were weakly maintained in AKT^D323H^. The weak salt bridge at the autoinhibitory interface of AKT^D323H^ renders its PH domain more susceptible to release than that of AKT^WT^. Taken together, our results reveal that the electrostatic interactions between the variable loops of the PH and the kinase domains contribute to the heterogeneity of the autoinhibitory interface of AKT.

### 2.3 AKT mutations disrupt the autoinhibitory interface to different extents

**FIGURE 5:**
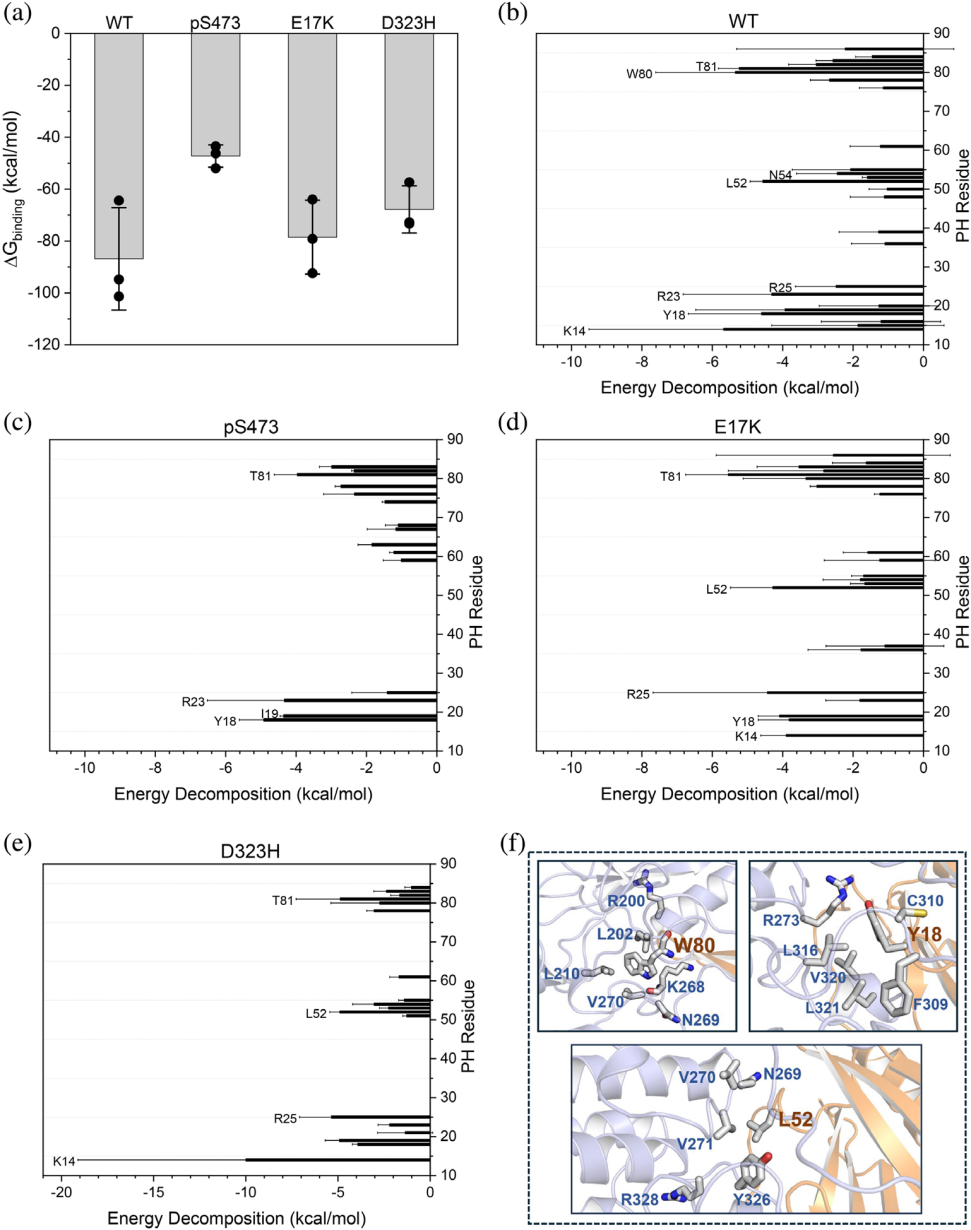
C‐terminal phosphorylation and mutations reduce the binding affinity between the PH and kinase domains. (a) Binding free energy between the kinase and PH domains for different systems. The binding free energy of each replica is represented by black dots. The binding energy shows that pS473 significantly reduces the binding affinity of the PH and kinase domains. (b‐e) Energy decomposition analysis of the contribution of the PH residues to the binding free energy for (b) AKT^WT^, (c) AKT^pS473^, (d) AKT^E17K^, and (e) AKT^D323H^. Residues in the PH domain displaying significant contributions (< −1.0 kcal/mol) to the binding free energy are identified and analyzed. Hydrophobic residues in the variable loops with significant contributions are marked. The energy calculations were based on three replicate simulations. (f) The hydrophobic interactions of W80, Y18, and L52 in AKT^WT^. These residues are in the variable loops, which contribute to the hydrophobic interactions at the autoinhibitory interface of AKT.

To assess the relative binding affinity between the PH and kinase domains, we calculated the binding free energy between the two domains (Figure 5a). Compared to AKT^WT^, phosphorylation and mutations cause a reduction in the binding affinity, with AKT^pS473^ showing the lowest affinity (2‐fold lower than AKT^WT^) between its PH and kinase domains. Other than electrostatic complementarity at the interface, hydrophobic interactions are the major driving force in protein stability (Zhou & Pang, 2018). To identify residues in the PH domain that contribute to the autoinhibited conformation of AKT, we decomposed the binding free energy on a per‐residue basis for each system (Figure 5b–e). As expected, charged residues such as K14, R23, and R25 contribute to the association of the PH with the kinase domain (Figure 4a). However, the contribution of the mutant residue K17 in AKT^E17K^ seems less important (>−1.0 kcal/mol and not labeled in Figure 5d), suggesting that the interaction of K17 with E298 of the activation loop contributes little to the binding affinity of the two domains. In AKT^WT^, hydrophobic residues with significant contributions to binding are in the variable loops of the PH domain, including Y18 and I19 in VL1, L52 in VL2, and W80 and T81 in VL3 (Figure 5b). However, the contributions of these residues differ in AKT^pS473^ (Figure 5c), AKT^E17K^ (Figure 5d), and AKT^D323H^ (Figure 5e).

E17K is the most reported AKT mutation, identified in 8% of breast, 6% of colorectal, 2% of ovarian and endometrial cancer patients (Carpten et al., 2007; Shoji et al., 2009). In addition to E17K, other rare mutations including K39N, P42T, L52R, and Q79K were identified in breast cancer patients (Yi et al., 2013), as well as engineered mutations Y18A (Bae et al., 2022), W80A and T81Y (Parikh et al., 2012), in the variable loops of the PH domain. However, not all these variants are activating mutations. Our energy decomposition analysis helps to elucidate to what extent the AKT mutations could relieve autoinhibition countering allosteric inhibitors. The most significant contribution to the binding free energy is from K14 (−5.6 kcal/mol) in AKT^WT^ (Figure 5b), and we use this value as a reference to quantify other residues. The charged residues R23 (−5.0 kcal/mol), and R25 (−2.9 kcal/mol) account for 89% and 52% of the contribution of K14, respectively, to the autoinhibitory interactions. Y18 is in VL1 of the PH domain and interacts with hydrophobic residues F309, L316, V320, and L321 (Figure 5f). Based on our energy decomposition of AKT^WT^, the contribution of Y18 (−4.6 kcal/mol) is greater than R25 (−2.9 kcal/mol), highlighting its importance in maintaining the autoinhibited conformation. Thus, Y18A can severely weaken the hydrophobic interactions between the PH and kinase domains, consistent with the experimental results implying that Y18A could relieve the PH domain‐mediated autoinhibition (Bae et al., 2022). In contrast, the contributions of K39 (−1.3 kcal/mol) and P42 (> −1.0 kcal/mol) in VL2 to the binding are less important (Figure 5b). Thus, the K39N and P42T mutations are not expected to disrupt the autoinhibitory interactions, in agreement with previous results showing that the two mutations did not activate AKT in biochemical or cellular assays and were most likely passenger mutations (Yi et al., 2013). While L52R is also in VL2, it interacts with hydrophobic residues N269, V270, V271 and Y326, as well as R328 in the kinase domain (Figure 5f). Its contribution (−4.6 kcal/mol) to the binding of the PH and the kinase domains is greater than R25 but less than K14 and R23. Consequently, L52R seems to moderate the disruption of the hydrophobic interactions between the PH and kinase domains and still respond to allosteric inhibitors, in line with previous studies (Yi et al., 2013; Yi & Lauring, 2016). The Q79K, W80A, and T81Y mutations occur in the shortest VL3 of the PH domain. The contribution of Q79 is predicted to be negligible; hence Q79K did not show resistance to AKT inhibitors (Yi & Lauring, 2016), but displayed enhanced affinity for PIP_3_ (Shi et al., 2014). In contrast, W80 (−5.3 kcal/mol) and T81 (−5.2 kcal/mol) provide a significant contribution to the autoinhibitory interactions. The bulky W80 interacts with hydrophobic residues (L202, L210, and V270) and charged residues (R200 and K268) in the N‐lobe of the kinase domain. W80A abolishes these interactions and consequently destabilizes the association between the PH and kinase domains. The introduction of another bulky tyrosine in T81Y, which is adjacent to W80, may produce steric hindrance and disrupt the binding of VL3 with the kinase domain. In this regard, W80A and T81Y could effectively relieve autoinhibition and render resistance to allosteric inhibitors, consistent with previous studies (Calleja et al., 2009; Green et al., 2008; Vivanco et al., 2014). Like electrostatic interactions, mutations can alter the relative contributions of those hydrophobic residues. For instance, in AKT^E17K^, T81 (−5.5 kcal/mol) contributes the most compared to other residues in the VL3 (Figure 5d). Collectively, our results reveal the pivotal role of the variable loops of the PH domain, particularly, the VL3, in modulating the hydrophobic interactions at the autoinhibitory interface. The extent of relief of autoinhibition and resistance to allosteric inhibitors by mutations is related to the contributions of those residues to the binding affinity between the PH and kinase domains of AKT.

### 2.4 Interaction between pS473 and Arg144 could allosterically trigger relief of autoinhibition

For AKT^pS473^, we have shown that there were negatively correlated motions between the PH and kinase domains (Figure 3b). Electrostatic interactions involving K14 and R25 were greatly weakened (Figures 4b and 5c), and the affinity between the two domains was significantly reduced (Figure 5a). We calculated the chemical shift perturbations (CSPs) for the PH residues of AKT^pS473^ with respect to AKT^WT^, and compared them with the experimental values obtained from ^15^N‐^1^H‐HQSC spectra of segmentally labeled full‐length AKT (Chu et al., 2020). The calculated CSPs are largely in qualitative agreement with the experimental results (Figure S12). The disordered loop regions show more peaks of magnitude higher than the standard deviation, indicating significant conformational dynamics. Thus, the CSPs for these disordered regions were not ambiguously assigned experimentally. In contrast, six β‐strands (β1, β2, β3, β5, β6, and β7) display more peaks of magnitude lower than the standard deviation, similar to the experimental observation. For β4, it shows CSPs with higher peaks than the standard deviation, suggesting that it undergoes large conformational changes. As a result, the CSPs for β4 cannot be ambiguously assigned by experiments.

**FIGURE 6:**
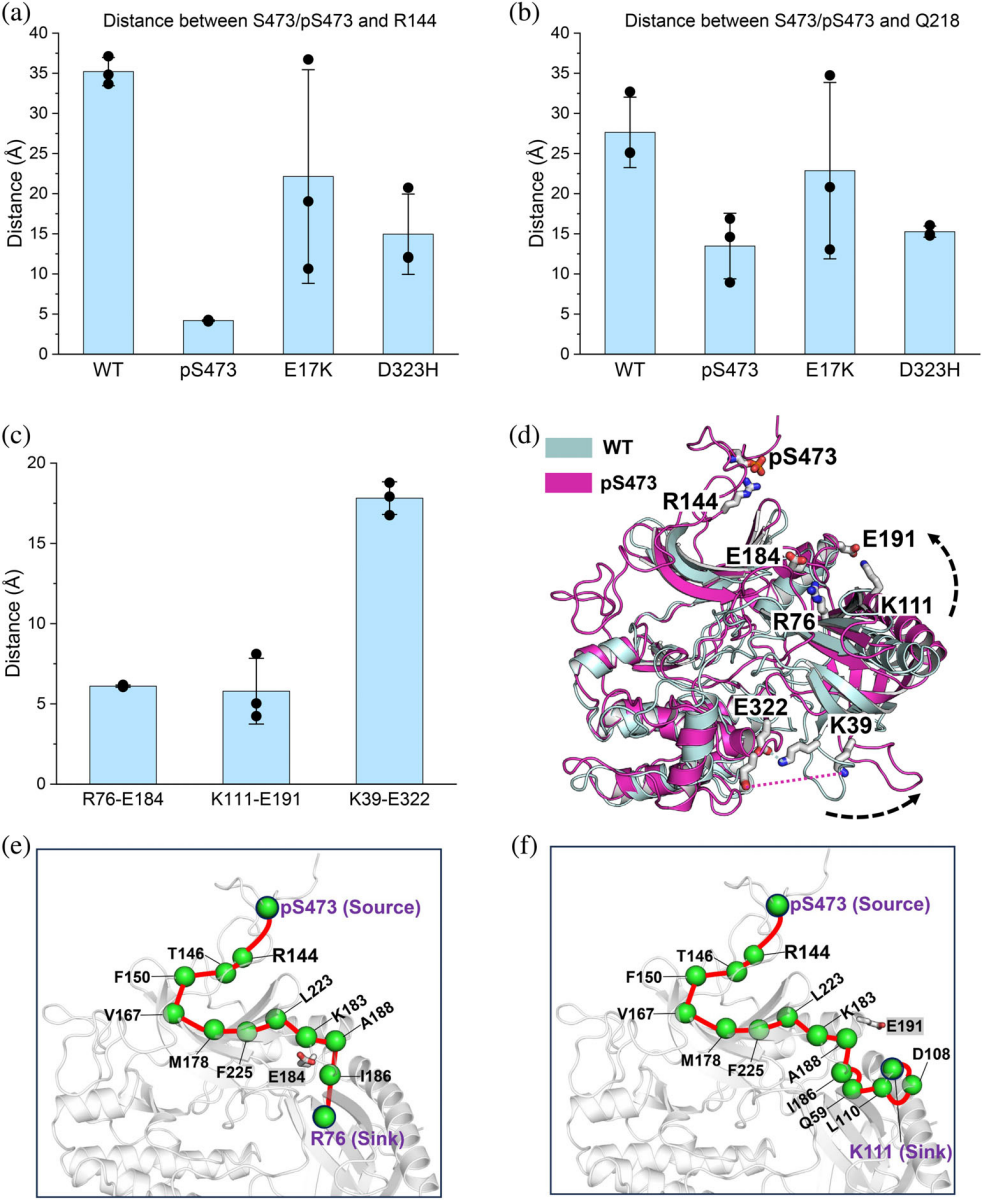
Interaction of pS473 with R144 allosterically displaces the PH domain from the kinase domain and consequently relieves autoinhibition. (a) The distance between S473/pS473 and R144 in different systems. (b) The distance between S473/pS473 and Q218 in different systems. (c) The distances of R76–E184, K111–E191, and K39–E322 in AKT^pS473^. The distance was calculated for each replicate simulation. The average value from each replicate simulation was denoted by black dots. (d) Structural superposition of AKT^WT^ with AKT^pS473^. E184 and E191 are in the N‐lobe of the kinase domain; E322 is in the C‐lobe of the kinase domain; K39 is in VL2 of the PH domain; R76 is in the vicinity of VL3; and K111 is in the C‐terminal region of the PH domain. Note that R76/K111 and K39 are on opposite sides of the PH domain. The allosteric pathway connects the source residue pS473 to the sink residues (e) R76 and (f) K111. For the definition of the distance and distance changes with simulation time, see Figures S13 and S14.

Next, we work out the allosteric communication between the phosphorylated C‐terminal tail and the PH domain to elucidate the underlying molecular mechanism. In our starting conformation, S473 is placed close to R144 and Q218 based on the available crystal structure and experimental data (see Methods). Over the simulations, the phosphorylated S473 (pS473), established a stable electrostatic interaction with R144, whereas the non‐phosphorylated S473 cannot maintain such an interaction (Figures 6a and S13 and S14). In all cases, we did not observe a stable interaction between S473/pS473 and Q218 (Figure 6b), probably because we used the full‐length structure of AKT in our study, whereas the interaction between D473 (mimicking phospho‐serine) and Q218 was observed in the crystal structure of the kinase domain only (Lin et al., 2012). Our results suggest that although the C‐terminal and linker of AKT are disordered, pS473 is capable of interacting with R144 which belongs to the basic patch of the linker region (including K140, K142, and R144). The interaction of pS473–R144 was proposed to induce a conformational change that relieves autoinhibition and leads to an active AKT (Chu et al., 2018; Cole et al., 2019), but the structural basis of the mechanism is unclear. Compared to AKT^WT^, we found that the K14–D323, R25–D323, and K39–E322 interactions disappeared when S473 was phosphorylated. On the other hand, R76, E85, and K111 established new interactions with E184, K307, and E191 of the kinase domain, respectively (Figure 4b). However, we did not observe coordinated changes (forming and breaking) in these salt bridges (Figure S15). The charged residues K14, E17, R23, R25, K39, and E49 are in or close to the variable loops of the PH domain. Residues E322, D323, and D235 are also in the loop region of the kinase domain. Thus, these salt‐bridge interactions are modulated by dynamic loop–loop interactions, rendering the heterogeneity of the autoinhibitory interface between the kinase and PH domains. These changes reduced contact between the PH domain and the C‐lobe of the kinase domain (specifically, E322 and D323), but enhanced contact between the PH domain and the N‐lobe of the kinase domain (specifically, E184 and E191) (Figure 6d). Consequently, the R76–E184 and K111–E191 interactions drive the tilting of the PH domain with respect to the kinase domain. The less populated E85–K307 interaction also contributes to this movement. In this way, the PH domain tends to move away from the kinase domain and can relieve autoinhibition.

To understand how pS473 allosterically promotes interactions of R76/K111 with the N‐lobe of the kinase domain, we constructed the allosteric pathways by dynamic network analysis and identified the most coupling residues (Materials and Methods). The pS473–R144 interaction is essential to trigger the allosteric effect through the N‐lobe of the kinase domain to the PH domain. From pS473 to R76, the allosteric path involves R144, T146, F150, V167, M178, F225, L223, K183, A188, and I186 to reach R76 (Figure 6e). Although E184 is not directly involved in the allosteric pathway, K183 and I186 in the vicinity of E184 help position it to interact with R76. The correlated motion from pS473 to K111 first shares the pathway to I186, then passes through Q59, L110, and D108 to reach K111 (Figure 6f). In this pathway, I186 and A188 that are adjacent to E191, and D108 and L110 that are adjacent to K111, can facilitate the interaction between K111 and E191. Taken together, our results provide the molecular rationale of how the pS473–R144 interaction allosterically triggers the relief of the PH domain from the kinase domain. Several residues identified in the pathways have been associated with various cancers. The COSMIC database (https://cancer.sanger.ac.uk/) (Sondka et al., 2024; Tate et al., 2019) reports the following mutations: R76H in two cases of brain cancer, E191K in three cases of large intestine/stomach adenocarcinoma, A188G in one case of lung cancer, and Q59L in one case of breast cancer. Their clinical significance remains uncertain based on ClinVar (https://www.ncbi.nlm.nih.gov/clinvar/) (Landrum et al., 2018). Further investigation is needed to elucidate the structural basis of how these reported mutations perturb AKT autoinhibition.

### 2.5 Mutations and C‐terminal phosphorylation differentially affect the accessibility of lipid binding sites in AKT

**FIGURE 7:**
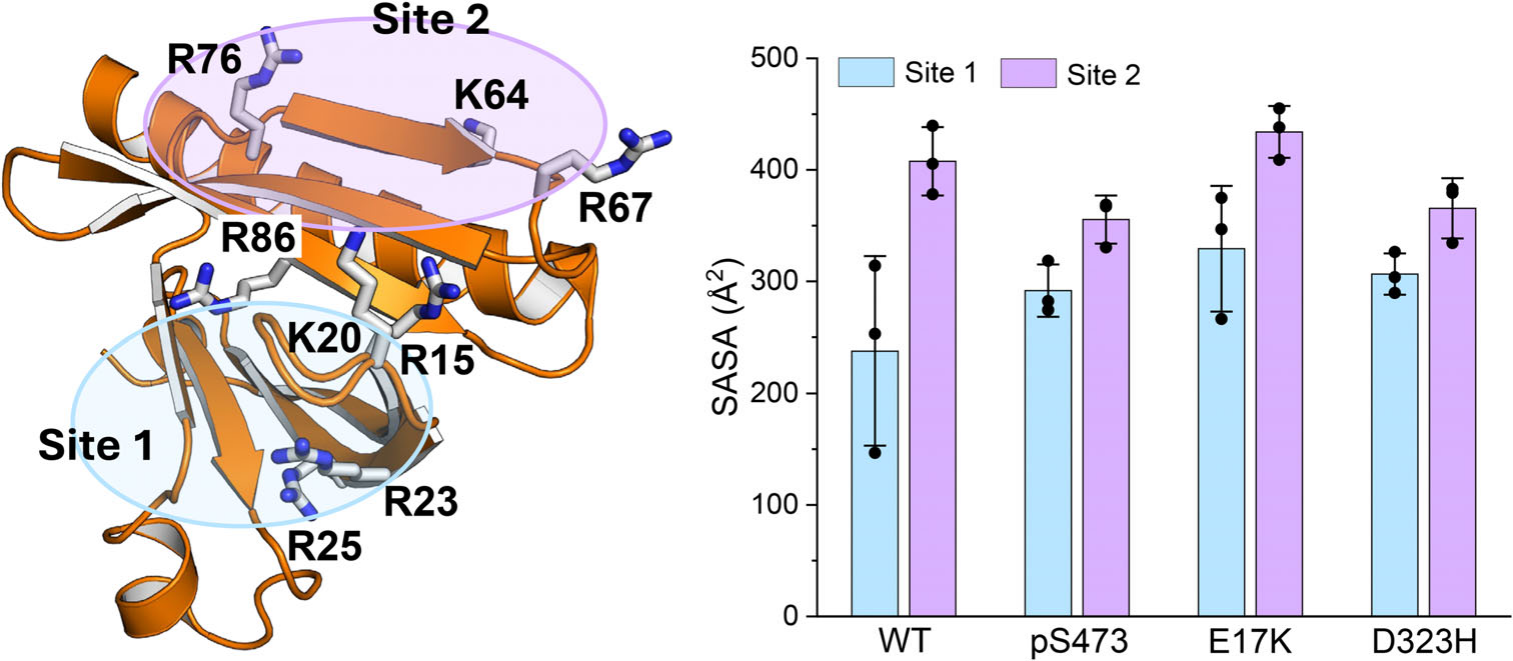
Different effects of phosphorylation and mutations on the two lipid binding sites in the PH domain of AKT. The canonical lipid binding site (Site 1) includes R15, K20, R23, and R25; and the second lipid binding site (Site 2) includes K64, R67, R76, and R86. The solvent accessible surface area (SASA) was calculated for those charged residues in each binding site. The SASA was calculated for each replica of each system (denoted as black dots).

Binding of the PH domain to PIP_3_ is required for recruitment of AKT to the membrane and its subsequent phosphorylation at T308 by PDK1 (Manning & Toker, 2017). The canonical lipid binding site of AKT in the PH domain involves R15, K20, R23 and R25 (Milburn et al., 2003). The interactions of R23 and R25 with E322 and D323 at the autoinhibitory interface prevent lipid binding (Figure 4a), especially by R25, as the R25A mutation precludes association with PIP_3_ (Rodriguez‐Escudero et al., 2009). A recent study combining MD simulations and molecular and cell biology identified a second lipid binding site involving K64, R67, R76, and R86, which acts cooperatively with the canonical binding site to recruit AKT to the membrane (Soteriou et al., 2025). To assess how the mutation and phosphorylation affect the accessibility of the two lipid‐binding sites, we calculated the solvent accessible surface area (SASA) of each binding site (Figures 7 and S16). Compared to AKT^WT^, the SASA of the canonical binding site (Site 1) increases 23%, 38%, and 29% in AKT^pS473^, AKT^E17K^, and AKT^D323H^, respectively, consistent with the reduced binding affinity between their kinase and PH domains (Figure 5a). The second lipid binding site is more accessible since it does not overlap with the autoinhibitory interface, with varying impacts of the mutations and C‐terminal phosphorylation on its accessibility. The SASA of the second binding site in AKT^pS473^ and AKT^D323H^ decreases 13% and 10%, respectively, but increases 6% in AKT^E17K^. The enhanced accessibility of both lipid binding sites in AKT^E17K^ enables this mutant to bind more strongly to membrane lipids, contributing to the constitutive activation of AKT. The opposing effects on the canonical and second lipid binding sites in both AKT^pS473^ and AKT^D323H^ suggest different mechanisms for modulating PH domain association with the plasma membrane and for allosterically activating AKT (Clark & Cullati, 2025; Ebner et al., 2017). Our results imply that mutations and phosphorylation may alter the autoinhibitory interface and influence AKT binding to the membrane in different ways.

## 3 DISCUSSION

Like many AGC kinases, AKT autoinhibition reduces spurious activation and signaling in the cytosol of unstimulated cells (Nussinov et al., 2018; Nussinov et al., 2020), and its allosteric inhibitors work by stabilizing the autoinhibited state. Lacking the variable loop‐mediated autoinhibitory interactions, PDK1 inhibitors bind to an allosteric PIF pocket, a site involved in protein–protein interactions. AKT activation is regulated by PDK1 and mTORC2, which respectively phosphorylate T308 and S473 of AKT (Manning & Cantley, 2007). How the mutations and phosphorylation of C‐terminal S473 relieve AKT autoinhibition has been unclear. A third site T450, which is constitutively phosphorylated by mTORC2, is required for AKT's stability (Facchinetti et al., 2008; Ikenoue et al., 2008), but may have minimal effects on AKT's transforming and signaling activities (Hart & Vogt, 2011).

**FIGURE 8:**
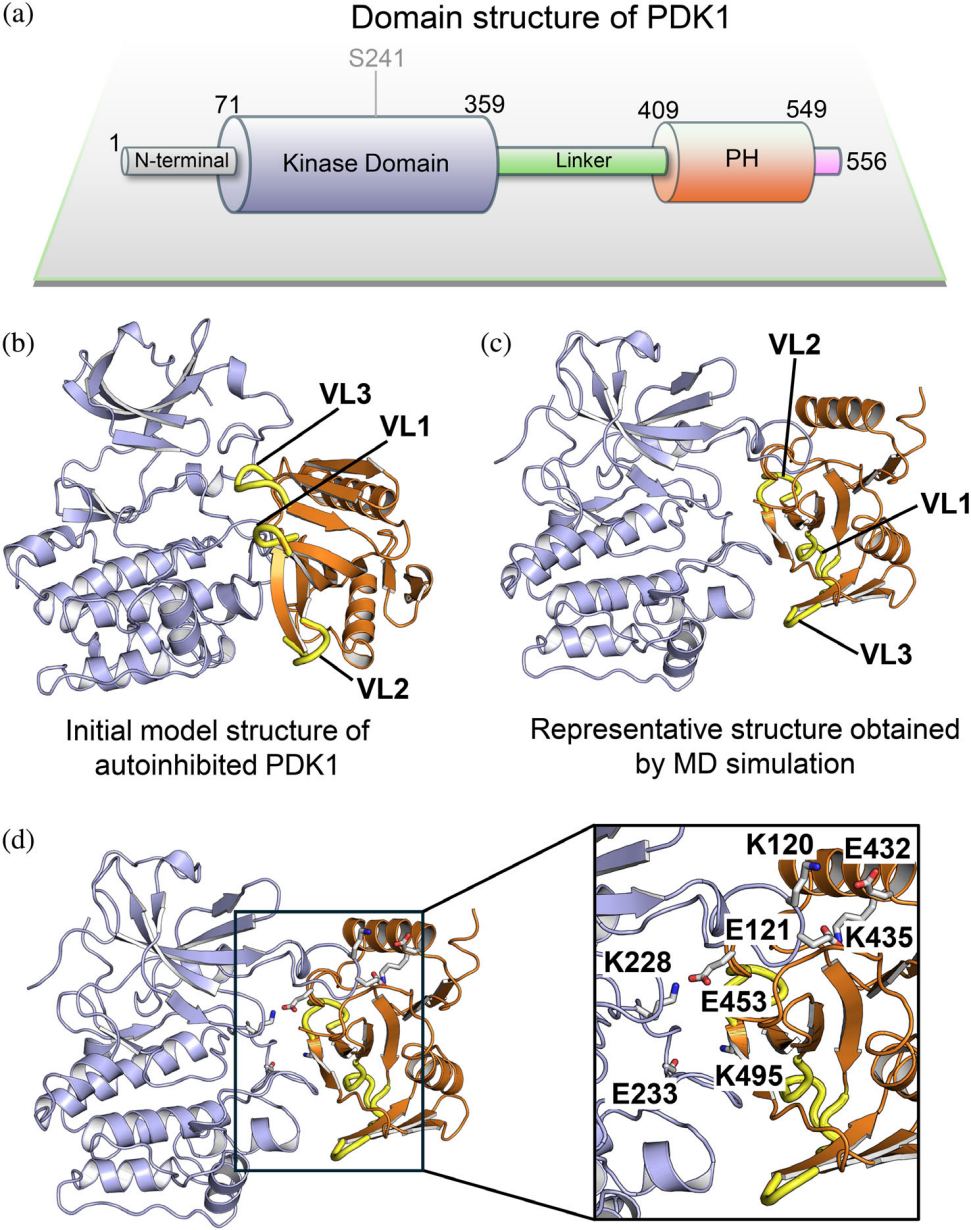
PDK1 lacks the variable loop‐mediated autoinhibitory interface between its PH and kinase domains. (a) Domain structure of PDK1 (UniProt identifier: O15530). PDK1 and AKT share similar kinase and PH domains. Phosphorylation of S241 on the activation loop is required for the activation of PDK1. (b) The starting conformation of autoinhibited PDK1. This structure displays a similar alignment of the kinase and PH domains to that observed in AKT. (c) The conformation of autoinhibited PDK1 obtained by MD simulations. The variable loops changed their relative positions at the autoinhibitory interface of PDK1. (d) The electrostatic interactions at the autoinhibitory interface of PDK1. No variable loop‐mediated interactions between the kinase and PH domains were observed.

We recently explored the conformational states of autoinhibited PDK1 by extensive simulations. PDK1 shares a similar kinase and PH domains with AKT (Figure 8a). We found that the autoinhibitory interactions between the PH and kinase domains were relatively stable when PDK1 adopted an AKT‐like autoinhibited conformation (Figure 8b) (Xu et al., 2024). To define the difference in the PH‐mediated autoinhibition between AKT and PDK1, we superimposed the PH domains of AKT and PDK1 and found that AKT has relatively longer and flexible variable loops (Figure S17). In the initial conformation of the autoinhibited PDK1, the positions of the variable loops of the PH domain resembled those of AKT; however, the PH domain of PDK1 underwent significant conformational changes over the simulations by shifting the relative positions of VL2 and VL3 (Figure 8c). For AKT, K14, R23, and R25 in VL1, as well as K39 in VL2, contributed to the association of the PH domain with the kinase domain (Figure 4a). In contrast, except for K495 in VL2, the interactions between the kinase domain and other residues of the PH domain stabilized the autoinhibitory interface of PDK1 (Figure 8d). In addition to electrostatic interactions, these variable loops in the PH domain of AKT also provide hydrophobic interactions that are critical for the maintenance of the autoinhibited state (Figure 5b). The absence of such specific variable loop‐mediated autoinhibition in PDK1 renders the unstable interaction between the PH and kinase domains and the autoinhibited states less populated, which ensures PDK1 effectively phosphorylates its substrate such as AKT in a lipid‐dependent manner, but phosphorylates S6K, SGK, and RSK kinases in a lipid‐independent manner.

These variable loops in the autoinhibited AKT provide a flexible binding interface for accommodating allosteric inhibitors (Figure S1). The binding of a covalent inhibitor, for instance, stabilized the interaction of E17 with R273 (PDB ID: 6S9W, Figure S2), but such an interaction was unstable in the absence of the inhibitor (Figure 4a). These variable loops also provide flexibility in AKT^E17K^. Mutant residue K17 in VL1 of the PH domain established an interaction with E298 in the activation loop of the kinase domain (Figure 4c), but the K17–E298 interaction contributed little to the binding between the PH and kinase domains (Figure 5d). The K17–E298 interaction is also observed in the recently determined crystal structure of AKT^E17K^ in complex with an allosteric inhibitor (PDB ID: 8UW2, Figure S2). These results suggest that E17K does not contribute significantly to relieving AKT autoinhibition. Instead, the potent oncogenic activation of AKT^E17K^ can be attributed to its enhanced affinity for membranes, increased catalytic activity in the absence of mTORC2, and greater resistance to pT308 dephosphorylation due to enhanced membrane binding, which keeps AKT^E17K^ in the active state (Bae et al., 2022). The different autoinhibitory interactions between AKT^WT^ and AKT^E17K^ are an inherent property of the heterogeneous autoinhibitory interface of AKT.

The mutations bias the conformational ensemble of AKT (Figure 2), still maintaining but mostly disrupting the autoinhibition. Our energy decomposition analysis indicated that oncogenic and engineered mutations in the variable loops modulate the autoinhibitory interactions. In addition to E17K, mutations like K39N and P42T seem unable to break the autoinhibitory interaction of AKT. In contrast, mutations such as L52R and Q79K, as well as engineered mutations including Y18A, W80A and T81Y, can significantly disrupt the autoinhibitory interface by weakening essential hydrophobic interactions and effectively relieve autoinhibition. D323 is not in any of the variable loops, but it interacts with R23 and R25 in the vicinity of VL1 (Figure 4a). The D323H mutation diminished both electrostatic and hydrophobic interactions between the PH and kinase domains, tending to release the autoinhibition (Figure 5e). These predictions are consistent with experimental results implying that K39N and P42T are passenger mutations. We labeled such mutations ‘latent driver’ mutations. These mutations behave like passenger mutations. However, together with other mutations, they can drive cancer development and drug resistance (Jang et al., 2023; Nussinov & Tsai, 2015; Yavuz et al., 2023). We also found that Y18A, L52R, W80A, T81Y, and D323H are activating mutations that are resistant to allosteric inhibitors. Thus, our unbiased simulations and energy analysis provide a structural basis for the functional consequences of AKT mutations.

**FIGURE 9:**
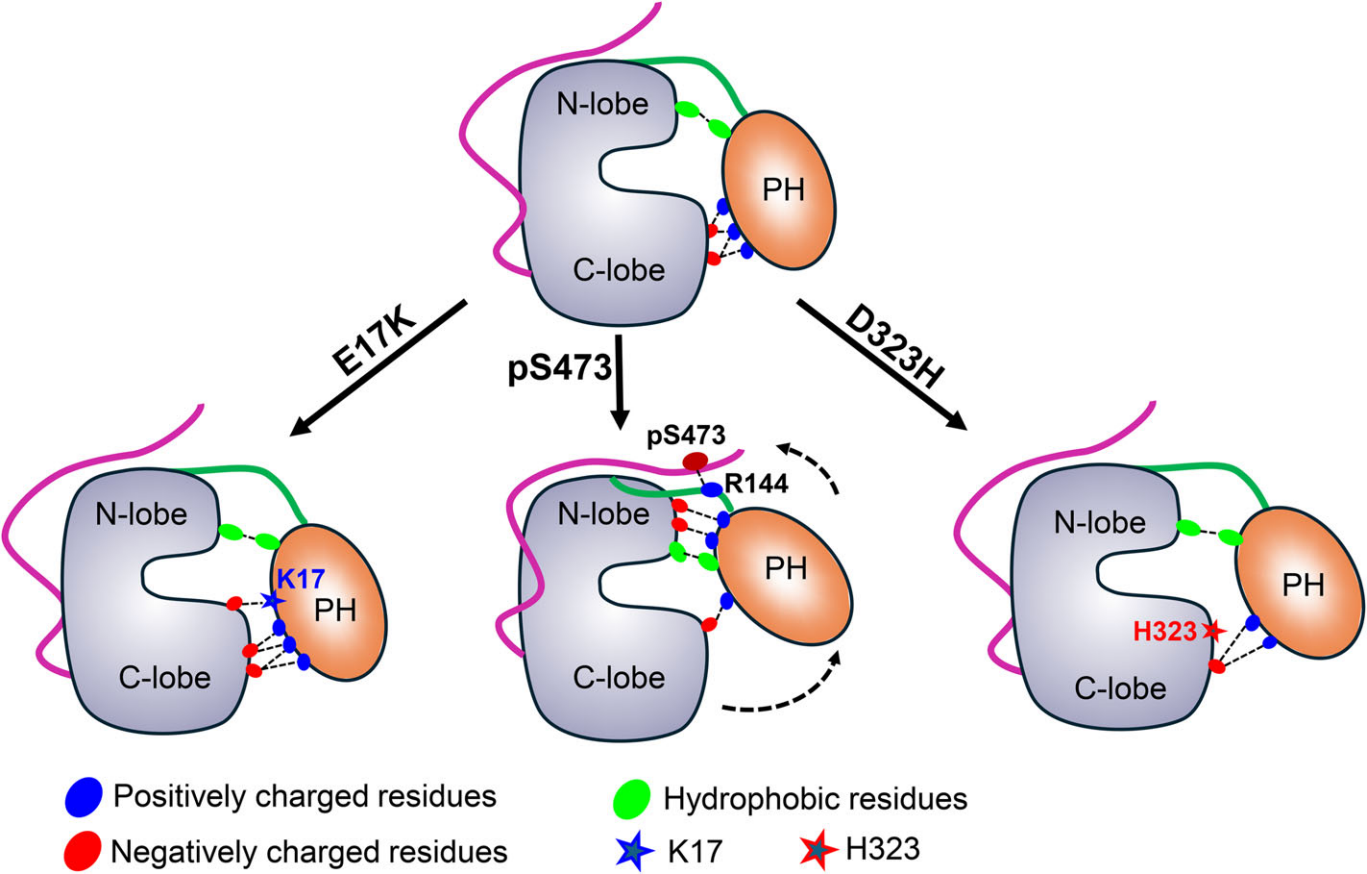
Schematic representation of the effects of C‐terminal phosphorylation and mutations on the conformational state of AKT. The wild‐type AKT remains in an autoinhibited conformation by intramolecular interactions between its kinase and PH domains. The autoinhibitory interface is modulated by electrostatic and hydrophobic interactions between the variable loops of the PH and the kinase domain. Phosphorylation of C‐terminal S473 allosterically triggers the interaction of the PH domain with the N‐lobe of the kinase domain while significantly mitigating the interaction of the PH domain with the C‐lobe of the kinase domain, promoting relief of autoinhibition. Oncogenic mutations E17K and D323H attenuate the association of the PH domain with the kinase domain but still maintain the autoinhibited conformation.

C‐terminal phosphorylation leads to distinct regulatory effects on AKT (Salguero et al., 2022). Phosphorylation of S473 is required for full activity. The interaction of pS473 with R144 was proposed to induce the release of autoinhibition, independent of the lipid (Cole et al., 2019). This requires the phosphorylated C‐terminal tail to bind to the N‐lobe of the kinase domain, enabling pS473 to be close to linker residue R144. Alterations in the linker reduced the pS473‐mediated activation of AKT (Chu et al., 2020). In our model of AKT^WT^, the C‐terminal tail fluctuates and cannot stably bind to the N‐lobe of the kinase domain (Figure 6). However, in an earlier structural model of full‐length autoinhibited AKT, the unphosphorylated C‐terminal tail (residues 457–480) bound the N‐lobe of the kinase domain. In that model, T450 was constitutively phosphorylated (Truebestein et al., 2021). In addition, the phosphorylation of S477 and T479 at the C‐terminal end may facilitate pS477/pT479 interaction with the activation loop by displacing the PH domain from the kinase domain (Chu et al., 2018). In our model of AKT^pS473^, the C‐terminal tail remains flexible but pS473 stably interacts with R144. We used this structural model to delineate the allosteric communication between the PH and kinase domains of AKT. We found that the pS473–R144 interaction triggers the R76–E184 and K111–E191 interactions, which tilt the PH domain relative to the kinase domain and disrupt the K39–E322 interaction that initially stabilized the association of VL2 with the kinase domain. We further identified the allosteric communication pathways from pS473 to R76 and K111. Notably, our results show that the position of R144 in the linker region is essential for the allosteric release of the PH domain, consistent with experimental data showing that increasing linker flexibility reduced AKT activation in AKT^pS473^ (Chu et al., 2020). This may explain why an altered linker failed to observe relief of autoinhibition in the absence of PIP_3_ (Lucic et al., 2018). Figure 9 summarizes the distinct effects of pS473, E17K, and D323H on the autoinhibited state.

Other PH domain binding proteins can also allosterically mediate the autoinhibitory interactions of AKT. In breast cancer cell lines, the PH domain of AKT binds to calmodulin, a ubiquitous calcium sensing molecule (Agamasu et al., 2017; Coticchia et al., 2009). The molecular mechanism of how calmodulin interacts with the free PH domain of AKT was elucidated in our prior study (Weako et al., 2021). The binding of calmodulin was proposed to attenuate the autoinhibitory interactions and facilitate AKT's translocation to the membrane (Agamasu et al., 2017). NMR chemical shift perturbation data suggested that calmodulin binds to two loops of the free PH domain, one loop connecting β6 and β7, and the other connecting β1 and β2 (Agamasu et al., 2015). The first calmodulin binding site overlaps with the second lipid binding site, and the second calmodulin binding site corresponds to VL1 (Figure 7). Since VL1 is buried in autoinhibited AKT, calmodulin is most likely to bind to the second lipid binding site first, which may allosterically expose the canonical lipid‐binding site in the PH domain, promote the release of autoinhibition, and consequently facilitate AKT translocation to the membrane, enhancing AKT activation.

**FIGURE 10:**
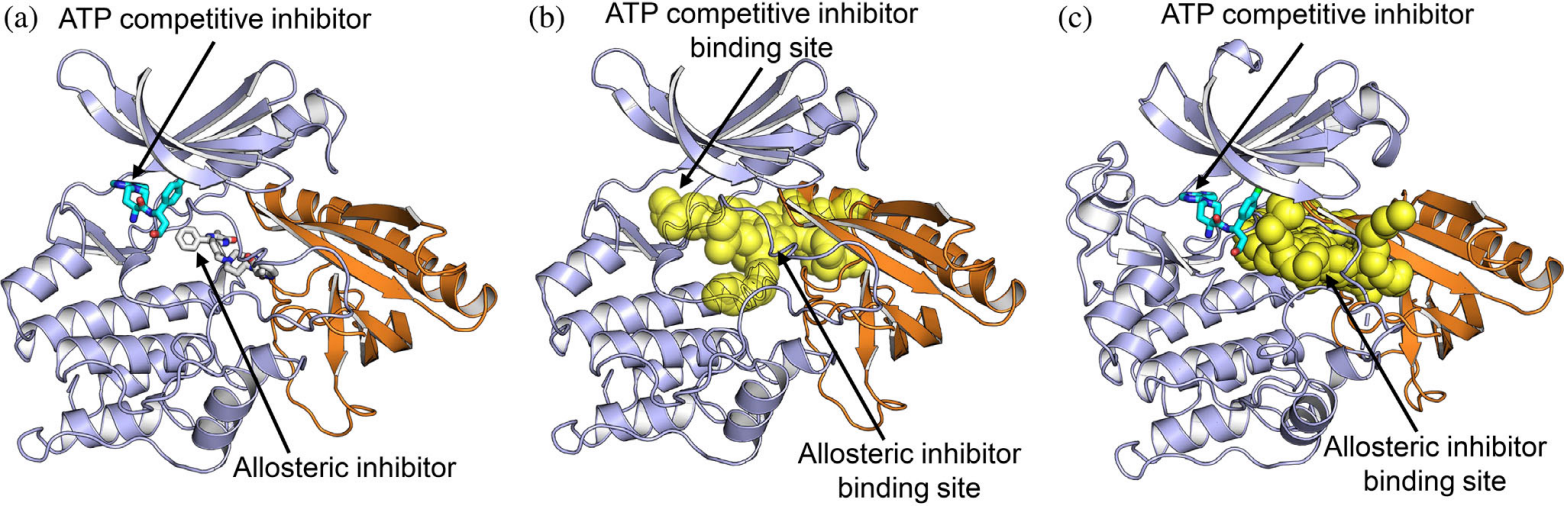
The binding pocket in the structure of autoinhibited AKT informs design of novel AKT inhibitors. (a) The binding sites for the ATP‐competitive and allosteric inhibitors in the structure of autoinhibited AKT. The binding site for the ATP‐competitive inhibitor (cyan) is obtained by superimposition the representative conformation of the AKT^WT^ with the crystal structure of AKT kinase domain in complex with capivasertib (Truqap) (PDB ID: 4GV1). The binding site for the allosteric inhibitor (white) is obtained by superimposition the same representative conformation of AKT with the crystal structure of AKT in complex with an allosteric inhibitor (PDB ID: 6S9W). (b) The binding pocket in the conformation of the autoinhibited AKT. The yellow spheres display the continuously accessible cavities, indicating that the ATP‐competitive and allosteric inhibitor binding sites are connected. For clarity, the linker and C‐terminal tail are not shown. (c) The ATP‐competitive and allosteric inhibitor binding sites in the crystal structure (PDB ID: 6S9W) are not connected. We used this structure to construct the full‐length autoinhibited structure of AKT. The continuously accessible cavities do not extend to the binding site for ATP‐competitive inhibitor.

Our results could inform AKT inhibitor design. Current AKT inhibitors can be classified into three categories: ATP‐competitive, allosteric, and AKT degrader (Landel et al., 2020; Shariati & Meric‐Bernstam, 2019; Tian et al., 2024). The ATP‐competitive inhibitor capivasertib (Truqap) (Figure 10a), in combination with fulvestrant (Faslodex, the estrogen receptor degrader) has been approved by the FDA for adult patients with breast cancer (Turner et al., 2023). The recent development of allosteric inhibitors specifically targeting AKT^E17K^ demonstrates their potential advantages in improving efficacy (Craven et al., 2025; Shrestha Bhattarai et al., 2022). In addition, AKT degraders, based on the structures of ATP‐competitive or allosteric inhibitors, were developed (You et al., 2020; Yu, Xu, Cahuzac, et al., 2022; Yu, Xu, Shen, et al., 2022). Our results show that the two inhibitor binding sites are not discrete but connected (Figure 10b). However, in the initial crystal structure, the binding site for the allosteric inhibitor is separated from the binding site for the ATP‐competitive inhibitor (Figure 10c). We further analyzed the binding pockets for the other crystal structures complexed with allosteric inhibitors and found that the two inhibitor binding sites could be connected even in the presence of allosteric inhibitors (Figure S18). This finding can provide insight into designing AKT inhibitors through (i) a combination of both ATP‐competitive and allosteric inhibitors; and (ii) the design of bitopic inhibitors that simultaneously bind to the ATP and allosteric binding sites. As shown in our prior study of mTOR kinase (Liu et al., 2024), the present unbiased conformation of AKT can also guide the optimization of bitopic inhibitors.

## 4 CONCLUSIONS

Here we address the structural basis of AKT autoinhibition and its release. Although it has been recognized that the PH domain mediates AKT's autoinhibition, our simulations reveal that the three variable loops in the PH domain contribute predominantly to the autoinhibitory interactions with the kinase domain. The lack of such variable loop‐mediated autoinhibition renders the autoinhibited PDK1 unstable, thus less populated. The heterogeneity of AKT's autoinhibitory interface makes it easily biased by allosteric inhibitors, mutations, and phosphorylation. It is not observed as sensitive to the oncogenic mutation E17K in VL1 but as vulnerable to mutations of hydrophobic residues in these variable loops. Further, we clarified how the interaction of phosphorylated S473 with R144 disrupts the autoinhibitory interface through allosteric communications between the N‐lobe of the kinase domain and the PH domain. These mechanistic insights are important for understanding how mutations, post‐translational modifications, and membrane binding control AKT activity and resistance to inhibitors. Especially, they point to *the essential role of conformational heterogeneity in regulating autoinhibition*. In these two kinases, which mediate signaling lipid PIP_3_ at the membrane, and are crucial at the top of the PI3K/PDK1/AKT cell growth signaling, *teetering autoinhibition stability with low kinetic barriers, is vital*. This is coupled with their large number of substrates (estimated over 100 for AKT; about 24 for PDK1) (He et al., 2021), including the recently identified N‐glycosyltransferase asparagine‐linked glycosylation 3 homolog (Navarro‐Traxler et al., 2025). Notably, not all autoinhibition mechanisms are unstable, as shown by the myristoylated N‐terminal region in Abl (Liu, Jang, et al., 2022). Abl, also with over 100 substrates, may have opted for the myristoylation mechanism since it not only inhibits kinase activity but also localizes Abl to membranes, with the myristoyl group acting as a dual‐role switch that competes with membrane insertion for activation (de Buhr & Grater, 2023).

## 5 MATERIALS AND METHODS

### 5.1 Construction of full‐length AKT in autoinhibition

Full‐length AKT (UniProt identifier: P31749) comprises the N‐terminal PH domain, the conserved kinase domain, an unstructured linker region between the PH and kinase domains, and a flexible C‐terminal regulatory domain (Figure 1a). We compared the crystal structures of autoinhibited AKT complexes available to date and chose the one with the defined αC‐helix in the kinase domain (PDB ID: 6S9W) (Quambusch et al., 2019) to model the full‐length structure of autoinhibited AKT (Figure S1). The inhibitor in the complex was removed. The crystal structure of the AKT kinase domain containing the C‐terminal residues S463–S477 (PDB ID: 4EKK) (Lin et al., 2012) was used to position S473 in the N‐lobe of the kinase domain of autoinhibited AKT by structural superimposition. The remaining C‐terminal residues were modeled as an unstructured chain. The linker region between the PH and kinase domains was modeled as unstructured with R144 being placed close to S473 (Figure 1b). Missing residues in the activation loop were also modeled as a loop. The full‐length structure of autoinhibited AKT was energy minimized and relaxed by performing short MD simulations in aqueous solution. In addition to AKT^WT^, the C‐terminal phosphorylated structure of AKT^pS473^ was constructed by phosphorylation of S473 (pS473). The structures of AKT^E17K^ and AKT^D323H^ were constructed by replacing E17 and D323 with lysine and histidine residues, respectively.

### 5.2 All‐atom MD simulations

We carried out MD simulations to explore the conformational ensemble of the autoinhibited AKT using the NAMD 2.14 package (Phillips et al., 2005; Phillips et al., 2020). We used the same simulation protocols as used in our previous studies (Jang et al., 2021; Liu et al., 2023; Liu et al., 2024; Liu, Jang, et al., 2022; Liu, Zhang, et al., 2022; Maloney et al., 2021; Xu et al., 2025; Zhang et al., 2019; Zhang et al., 2021). We represented the full‐length AKT structure using the updated and modified version of the CHARMM all‐atom additive force field (version 36 m) (Huang et al., 2017; Klauda et al., 2010) and solved the protein in a cubic water box filled with TIP3P water molecules. The minimum distance between the protein and the edge of the water box was 15 Å. Each system was neutralized by adding 100 mM NaCl. The Nosé‐Hoover Langevin piston pressure control and Langevin temperature control were used to maintain the pressure at 1 atm and the temperature at 310 K, respectively (Braga & Travis, 2005; Martyna et al., 1994). The long‐range electrostatic interactions were calculated using the particle mesh Ewald (PME) method (Darden et al., 1993), with the PME grid spacing of 1.0 Å and PME interpolation order of 6. We calculated the van der Waals (vdW) interactions using a switching function with a twin cutoff of 10 Å and 12 Å, and applied the SHAKE algorithm to constrain the motion of bonds involving hydrogen atoms (Ciccotti & Ryckaert, 1986). We used an integration time step of 2 fs. Each system was first energy minimized for 20,000 steps and then equilibrated in the NVT ensemble (constant volume and temperature at 310 K) for 5 ns with Cα atoms restrained using harmonic restraints. The production run was performed in the NPT ensemble (constant pressure at 1 atm and temperature at 310 K) without any restraints and lasted for 1 μs. Three replicas were generated for each system, and no significant difference was observed between the replicas. The last half of each trajectory was used for analysis.

### 5.3 Conformational dynamic analysis

We first performed PCA to investigate the large‐scale motions of the protein dynamics using the Bio3D software (Grant et al., 2006; Grant et al., 2021). All conformations were aligned to the initial conformations in terms of the coordinates of the Cα atoms, excluding the disordered C‐terminal and linker regions. A covariance matrix was constructed from the atomic fluctuations of the Cα atoms around their mean positions, which captures the correlated motions within the protein during MD simulations. The principal components (PCs) were then computed using the eigenvalue decomposition of the covariance matrix. The eigenvalues represent the variance displayed by an individual PC, and the eigenvectors describe the direction of the motion (Groth et al., 2013). Typically, the first few PCs capture most of the protein's collective motion. Here, we focus on the first two PCs and calculate the contribution of individual residues to the first PC to identify key regions of the protein involved in large‐scale conformational motions.

We then performed DCCM analysis to identify the residues maintaining the cross correlations, that is, the extent to which the residue fluctuations of a protein are correlated with one another. The dynamical cross‐correlation $C_{\mathrm{ij}}$ between the *i*th and *j*th Cα atoms is defined as

$$C_{\mathrm{ij}}=\frac{\left\langle ∆r_{i}\left(t\right)∆r_{j}\left(t\right)\right\rangle }{\sqrt{\left\langle ∆r_{i}{\left(t\right)}^{2}\right\rangle \;\left\langle ∆r_{j}{\left(t\right)}^{2}\right\rangle }},$$

where ***r***_*i*_ *(t)* denotes the vector of the *i*th atom's coordinates as a function of time *t*, $\left\langle \cdot \right\rangle$ denotes the time ensemble average and $∆r_{i}\left(t\right)=r_{i}\left(t\right)-\left\langle r_{i}\left(t\right)\right\rangle$. The *C*_*ij*_ value is between −1 and 1, where positive values indicate that the residues move in the same direction in most of the frames; negative values indicate that the residues move in opposite directions in most frames; and a value close to zero indicates the motion is uncorrelated.

We used dynamic network analysis (Sethi et al., 2009) to construct the allosteric communication pathway between the C‐terminal tail and the PH domain. Within the network, the Cα atom of each residue represents a single node, and an edge connects two nodes if they are in contact (<4.5 Å) for at least 75% of the frames analyzed. To reduce the number of trivial paths, neighboring nodes are not considered to be in contact. The network was weighted by the probability of information transfer across the edge between nodes *i* and *j*, that is, *W*_*ij*_ = −log(|*C*_*ij*_|) where *C*_*ij*_ denotes the cross correlation as defined above. The shortest path indicates that the coupling is strongest between the selected two nodes (source and sink residues), representing the optimal pathway involving key residues in allosteric signal transduction. The dynamic network analysis was analyzed using the NetworkView plugin in VMD (Humphrey et al., 1996). For each system, we analyzed the second half of the trajectories from three replicate simulations.

### 5.4 Binding free energy calculation and decomposition

We calculated the binding free energy between the PH and kinase domains of AKT in terms of the molecular mechanics energies combined with generalized Born and surface area solvation (MM/GBSA) method (Genheden & Ryde, 2015; Wang et al., 2019). The averaged binding free energy $\left\langle ∆G_{b}\right\rangle$ is calculated as a sum of the gas‐phase molecular mechanics energy $\left\langle ∆E_{\mathrm{MM}}\right\rangle$, the solvation energy contribution $\left\langle ∆G_{\mathrm{sol}}\right\rangle$, and the entropy contribution –*T*Δ*S*,

$$\left\langle ∆G_{b}\right\rangle =\left\langle ∆E_{\mathrm{MM}}\right\rangle +\left\langle ∆G_{\mathrm{sol}}\right\rangle -T∆S,$$

where angle bracket denotes an average along the MD trajectory. The gas‐phase molecular mechanics energy is a sum of the internal/bonded energy, the electrostatic energy, and the vdW interaction energy,

$$∆E_{\mathrm{MM}}=∆E_{\text{inter}}+∆E_{\text{elec}}+∆E_{\mathrm{vdW}}.$$

The solvation energy contribution can be divided into the electrostatic contribution and the non‐polar contribution,

$$\Delta G_{\mathrm{sol}}=\Delta G_{\mathrm{sol}}^{\text{elec}}+\Delta G_{\mathrm{sol}}^{\text{nonpolar}},$$

where the electrostatic contribution was calculated using the generalized Born model (Onufriev et al., 2004). The nonpolar contribution is obtained using an equation

$$\Delta G_{\mathrm{sol}}^{\text{nonpolar}}=\gamma \cdot \text{SASA}+\beta ,$$

where *SASA* denotes the solvent‐accessible surface area (Wang & Kollman, 2000), with a set of the surface tension parameters, *γ* = 0.00542 and the constant *β* = 0.92.

The entropy contribution, *T*Δ*S*, to the binding free energy was calculated by normal mode analysis (Srinivasan et al., 1998). The change in binding free energy due to the complex formation is calculated using the equation,

$$\Delta G_{b}=\Delta G_{b}^{\mathrm{KD}-\mathrm{PH}}-\left(∆G_{b}^{\mathrm{KD}}+∆G_{b}^{\mathrm{PH}}\right).$$

where KD‐PH represents the complex formed by the PH and kinase domains only. The binding free energy was calculated based on a set of conformational snapshots taken from the MD simulations. A total of 2500 snapshots taken from the latter half of each simulation were used to calculate the gas‐phase and solvation energy contributions, whereas a total of 50 snapshots were used to calculate the entropy contribution due to its heavy computational cost. For each system, the final Δ*G*_*b*_ was averaged over the results of three replica simulations. The binding free energy was calculated using the Perl script mm_pbsa.pl. with default parameters in Amber22 program (Case et al., 2023). The free energy contributions to the binding free energy were further decomposed on a per‐residue basis. Residues in the PH domain displaying significant contributions (<−1.0 kcal/mol) to the binding free energy were identified and analyzed. Note that the Δ*G*_*b*_ values estimated with the MM/GBSA method can be much lower than the experimental data and thus only the relative values are reasonable (Wang et al., 2019).

## AUTHOR CONTRIBUTIONS

**Liang Xu:** Methodology; investigation; writing – original draft; software; formal analysis; writing – review and editing; validation. **Meryem Eren:** Methodology; software; formal analysis; writing – original draft; investigation. **Jackson Weako:** Methodology; software; formal analysis; writing – original draft; investigation. **Hyunbum Jang:** Conceptualization; methodology; writing – review and editing. **Ozlem Keskin:** Conceptualization; writing – review and editing; supervision. **Attila Gursoy:** Conceptualization; supervision; writing – review and editing. **Ruth Nussinov:** Conceptualization; funding acquisition; writing – review and editing; supervision.

## CONFLICT OF INTEREST STATEMENT

The authors declare no competing financial interest.

## Supporting information


**DATA S1.** Supporting Information.

## Data Availability

The structure files and trajectories generated from MD simulations were deposited to Zenodo and can be accessed via the link: https://doi.org/10.5281/zenodo.17187387.
