# Supplementary material for: The structural heterogeneity of AKT autoinhibition
Source: Protein Sci. 2025 Dec 23;35(1):e70420. doi: 10.1002/pro.70420 (PMC12724016; doi:10.1002/pro.70420)
Supplement: Supplementary file 1 — DATA S1. Supporting Information. [file PRO-35-e70420-s001.pdf]

Supplemental Materials

# The structural heterogeneity of AKT autoinhibition

Liang Xu,[1,#] Meryem Eren,[2,#] Jackson Weako,[3,#] Hyunbum Jang,[1] Ozlem Keskin,[4,*] Attila Gursoy,[5,*] and Ruth Nussinov,[1,6,*]

[1]Computational Structural Biology Section, Frederick National Laboratory for Cancer Research in the Cancer Innovation Laboratory, National Cancer Institute, Frederick, MD 21702, U.S.A.
[2]Department of Molecular Biology and Genetics, Koç University, Istanbul 34450, Turkey
[3]Computational Science and Engineering Program, Koç University, Istanbul 34450, Turkey
[4]Department of Chemical and Biological Engineering, Koç University, Istanbul 34450, Turkey
[5]Department of Computer Engineering, Koç University, Istanbul 34450, Turkey
[6]Department of Human Molecular Genetics and Biochemistry, Sackler School of Medicine, Tel Aviv University, Tel Aviv 69978, Israel

[#]These authors contributed equally to this work

[*]Corresponding authors:
Ozlem Keskin, okeskin@ku.edu.tr, Department of Chemical and Biological Engineering, Koç University, Istanbul 34450, Turkey
Attila Gursoy, agursoy@ku.edu.tr, Department of Computer Engineering, Koç University, Istanbul 34450, Turkey
Ruth Nussinov, NussinoR@mail.nih.gov, Frederick National Laboratory for Cancer Research, Frederick, MD 21702, U.S.A.

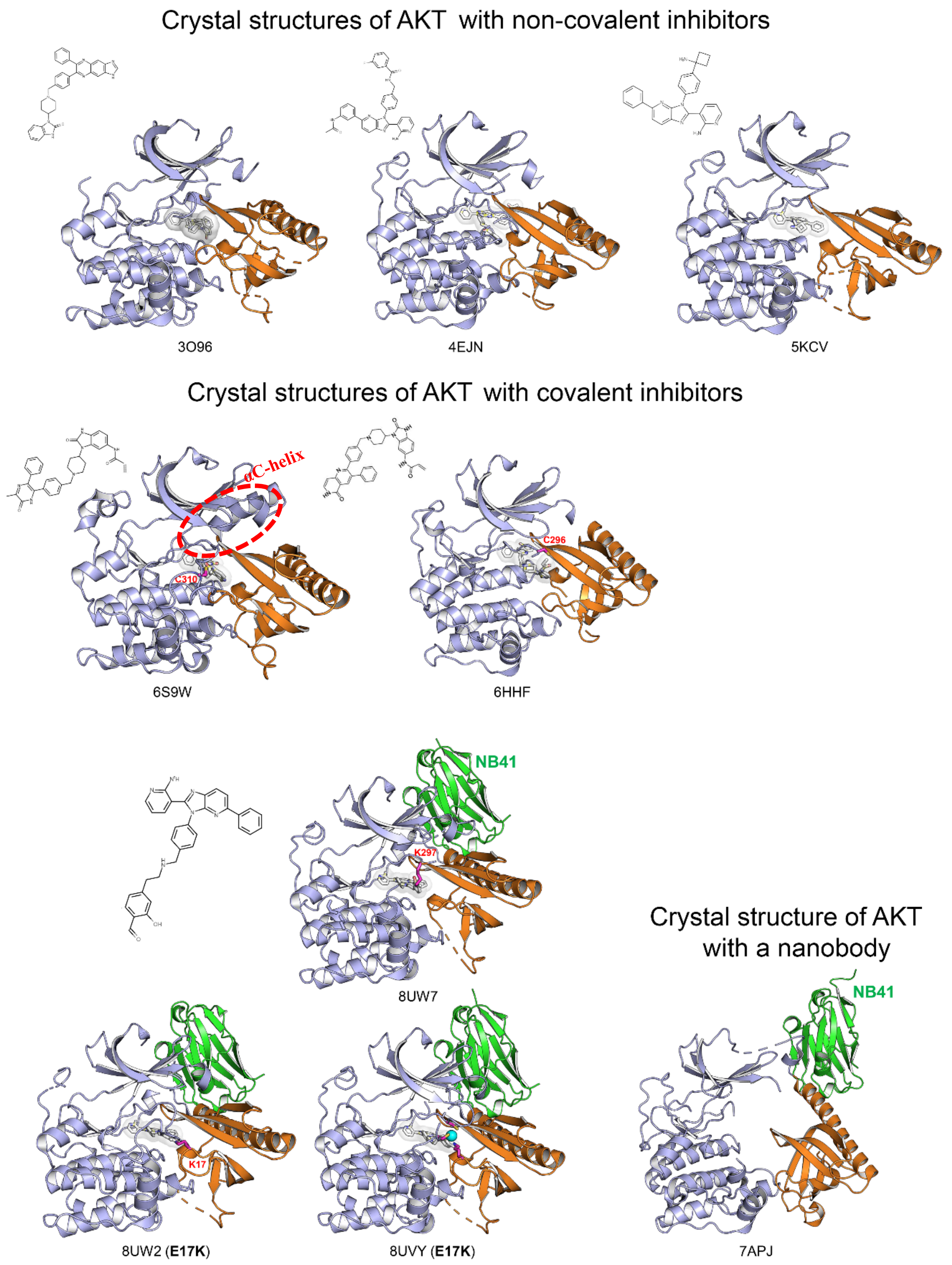


**Fig. S1. The crystal structures of AKT with different allosteric inhibitors.** The PH domain and kinase domain are colored orange and blue, respectively. The nanobody NB41 is colored green.

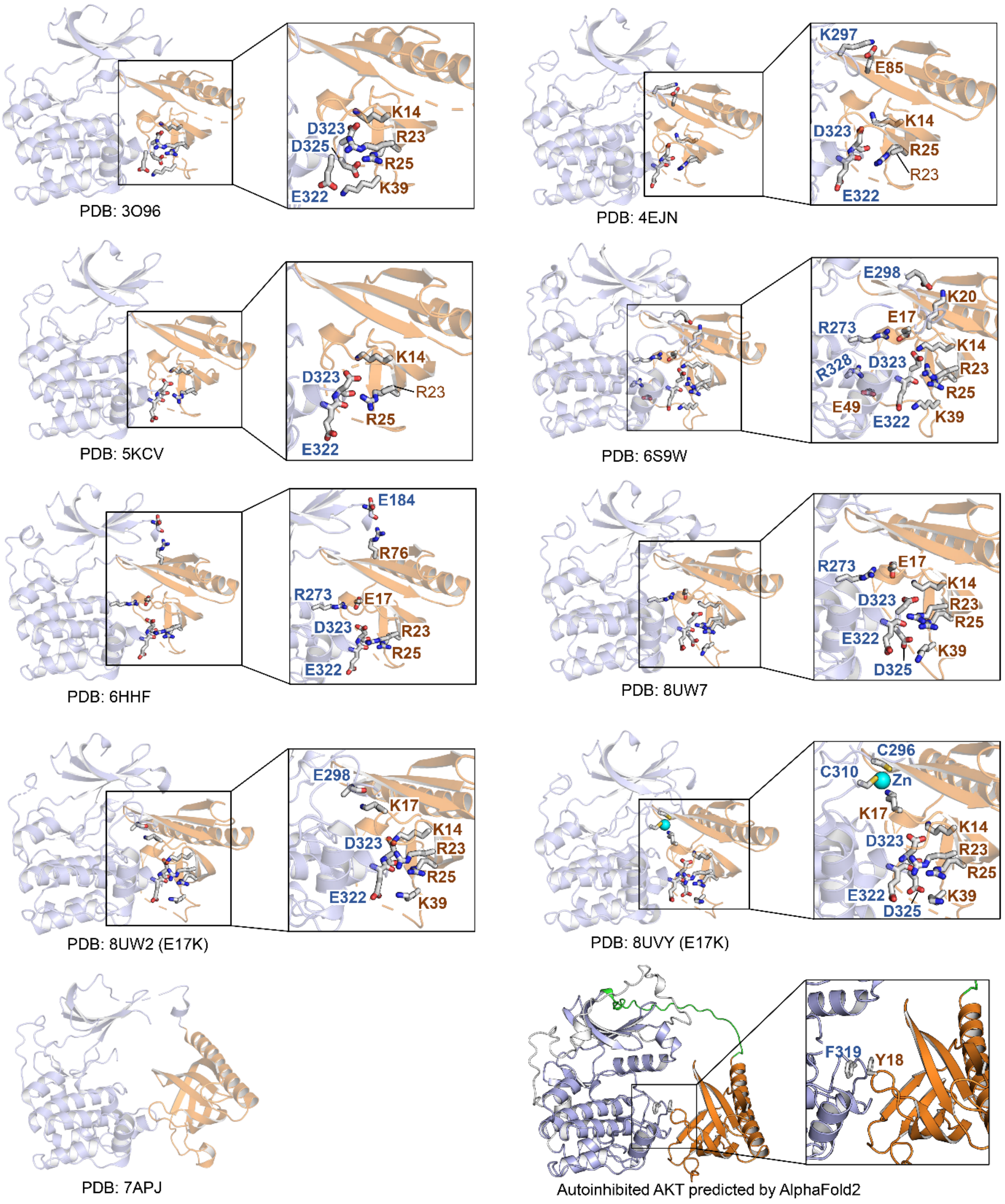


**Fig. S2. The electrostatic interactions at the autoinhibitory interface of available crystal structures of AKT bound with allosteric inhibitors.** For comparison, the structure of full-length AKT predicted by AlphaFold2 is also shown where hydrophobic interaction between F319 and Y18 is shown. No electrostatic interaction is present in the crystal structure of 7APJ.

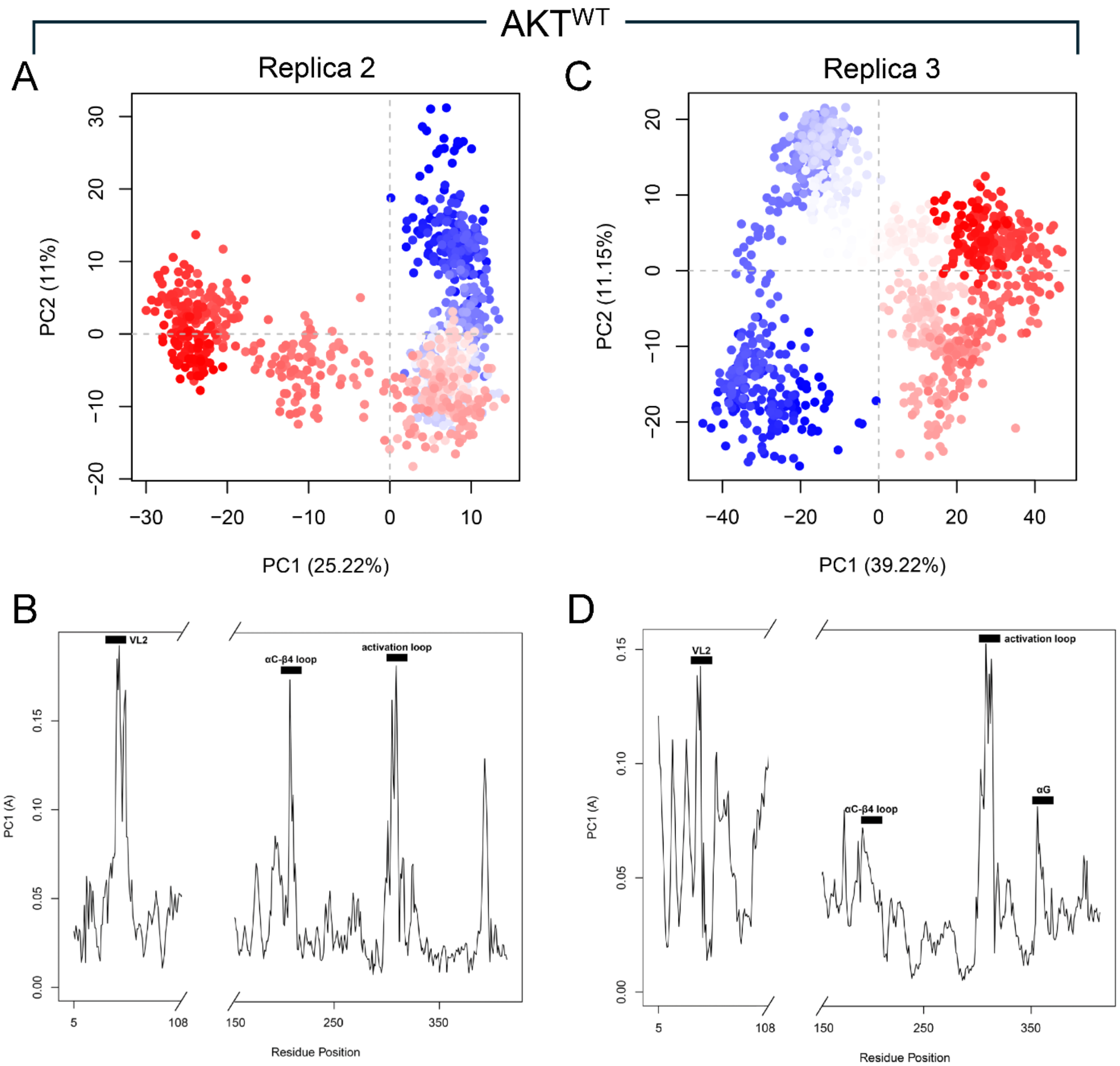


**Fig. S3. Principal component analysis (PCA) for the other two replicate simulations of AKT$^{WT}$.** The percentage of the first two components (PC1 and PC2) accounting for the total variance is shown in parentheses. (**A** and **C**) PCA results for replicate 2 and replicate 3 simulations of AKT$^{WT}$. Each dot represents one snapshot of the trajectory and the color from blue to red represents the trajectory frame from beginning to end. (**B** and **D**) PC1 loading plots for replicate 2 and replicate 3 simulations of AKT$^{WT}$. The regions with high conformational variability have more contribution to the PC1. The linker and C-terminal regions are disordered and excluded for PCA.

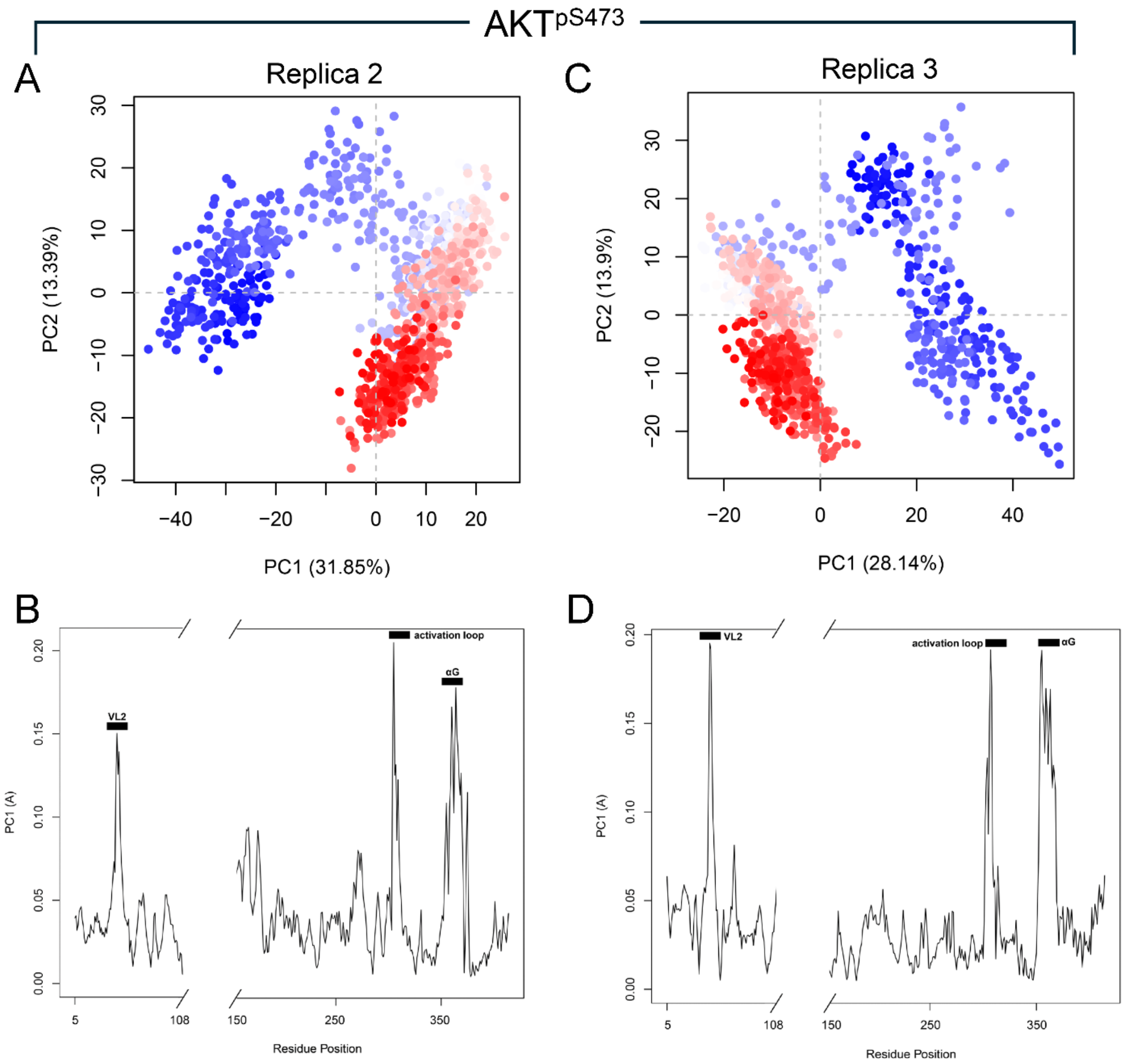


**Fig. S4. Principal component analysis (PCA) for the other two replicate simulations of AKT$^{pS473}$.** The percentage of the first two components (PC1 and PC2) accounting for the total variance is shown in parentheses. (**A** and **C**) PCA results for replicate 2 and replicate 3 simulations of AKT$^{pS473}$. Each dot represents one snapshot of the trajectory and the color from blue to red represents the trajectory frame from beginning to end. (**B** and **D**) PC1 loading plots for replicate 2 and replicate 3 simulations of AKT$^{pS473}$. The regions with high conformational variability have more contribution to the PC1. The linker and C-terminal regions are disordered and excluded for PCA.

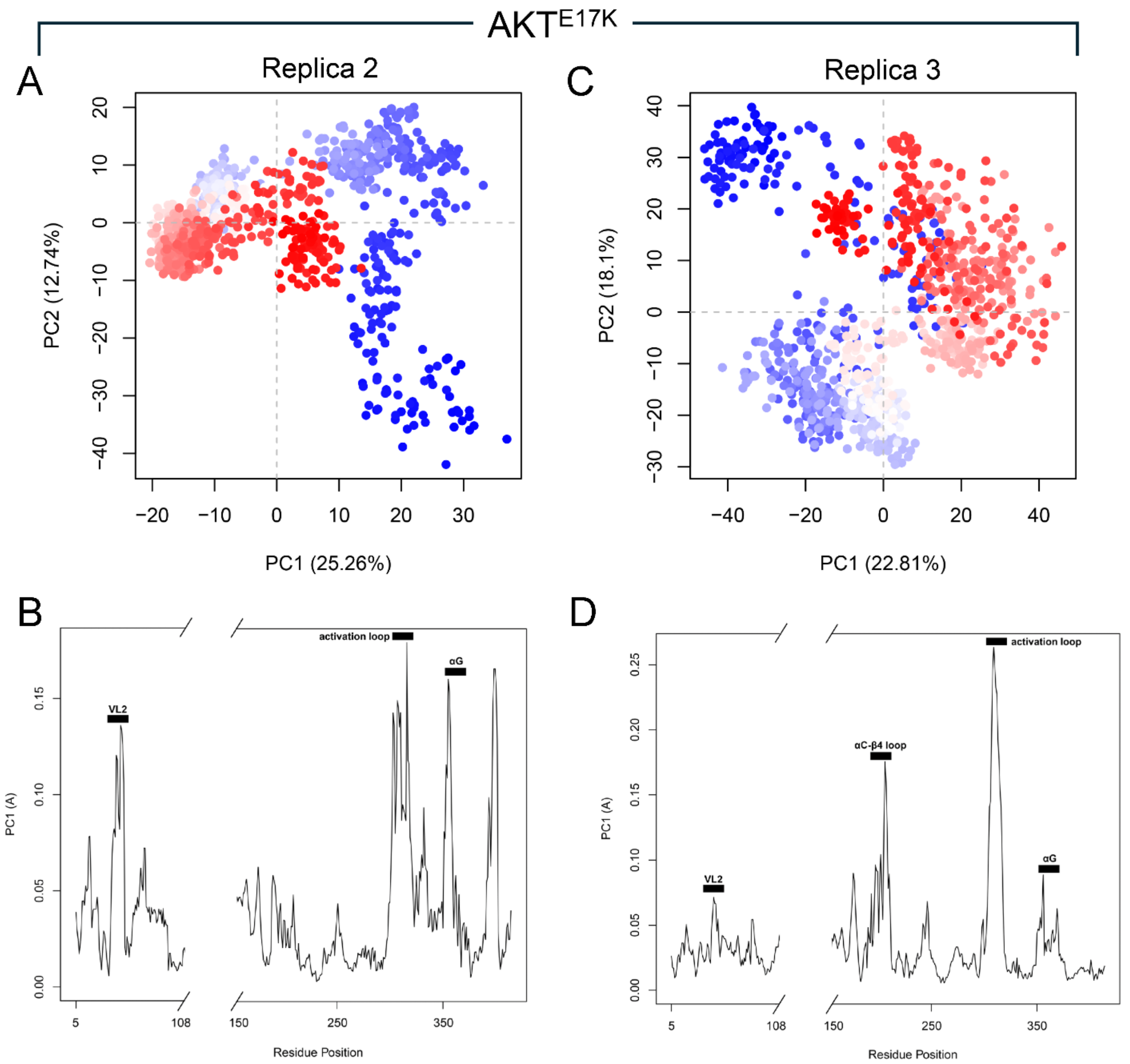


**Fig. S5. Principal component analysis (PCA) for the other two replicate simulations of AKT$^{E17K}$.** The percentage of the first two components (PC1 and PC2) accounting for the total variance is shown in parentheses. (**A** and **C**) PCA results for replicate 2 and replicate 3 simulations of AKT$^{E17K}$. Each dot represents one snapshot of the trajectory and the color from blue to red represents the trajectory frame from beginning to end. (**B** and **D**) PC1 loading plots for replicate 2 and replicate 3 simulations of AKT$^{E17K}$. The regions with high conformational variability have more contribution to the PC1. The linker and C-terminal regions are disordered and excluded for PCA.

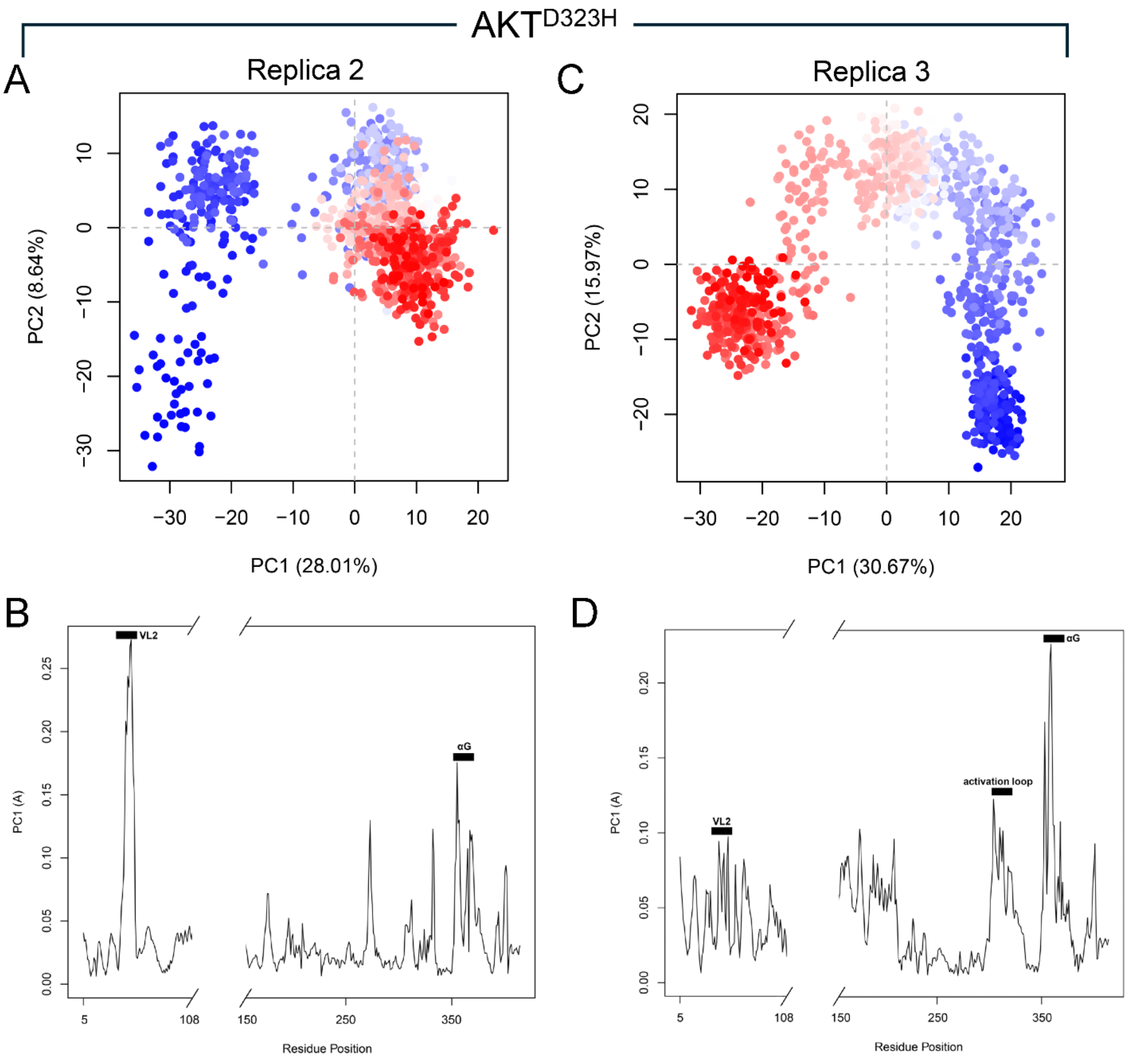


**Fig. S6. Principal component analysis (PCA) for the other two replicate simulations of AKT$^{D323H}$.** The percentage of the first two components (PC1 and PC2) accounting for the total variance is shown in parentheses. (**A** and **C**) PCA results for replicate 2 and replicate 3 simulations of AKT$^{D323H}$. Each dot represents one snapshot of the trajectory and the color from blue to red represents the trajectory frame from beginning to end. (**B** and **D**) PC1 loading plots for replicate 2 and replicate 3 simulations of AKT$^{D323H}$. The regions with high conformational variability have more contribution to the PC1. The linker and C-terminal regions are disordered and excluded for PCA.

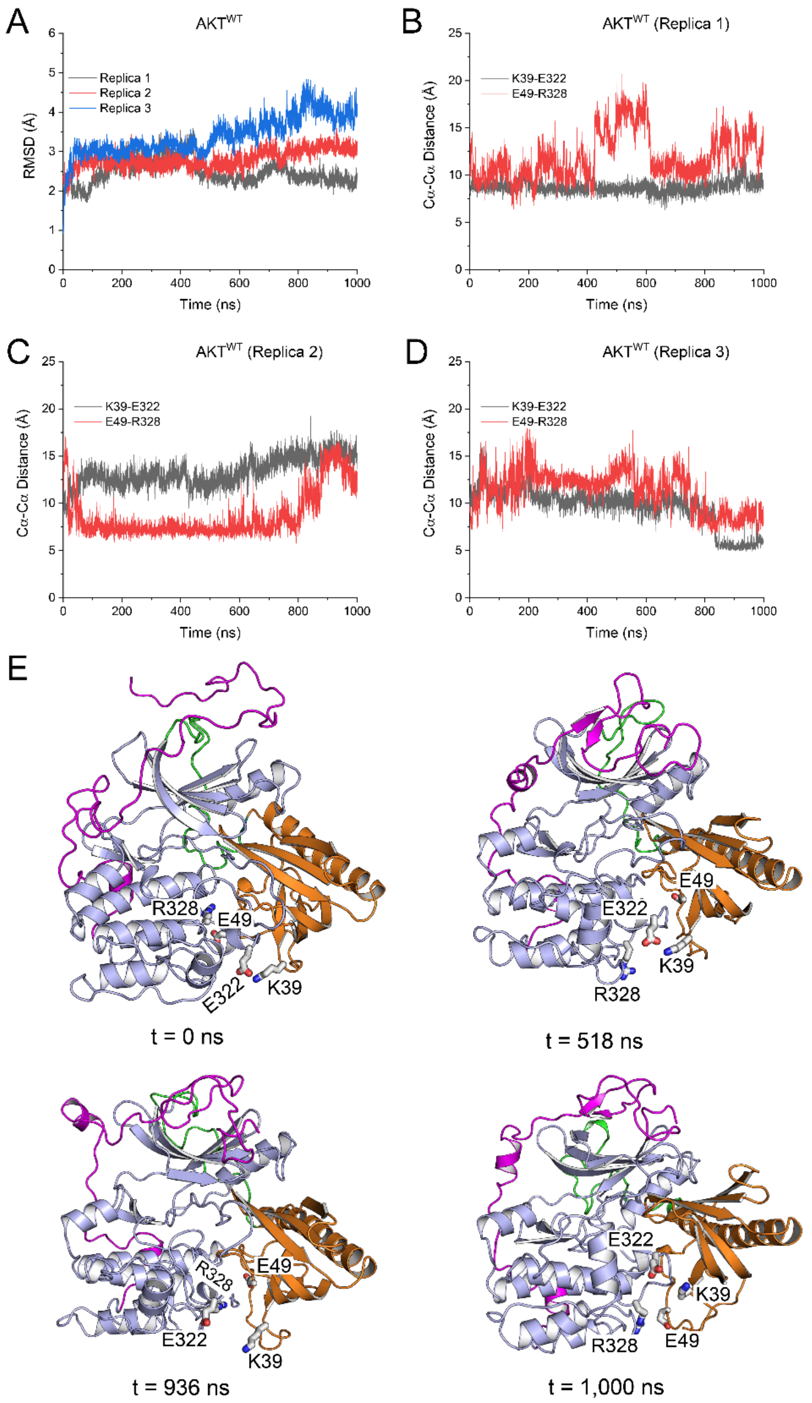


**Fig. S7.** (**A**) The root-mean-square deviation (RMSD) of Cα atoms of AKT$^{WT}$ during the simulations. The linker and C-terminal region are excluded for structural alignment. (**B–D**) The changes in the distance of K39-E322 and E49-R328 in three replicate simulations. K39 and E49 are in the variable loop 2 which undergoes substantial conformational changes. (**E**) Structural snapshots taken from the trajectory of replica 1 at different simulation time scales, showing the conformational dynamics of the variable loop 2. The kinase domain, PH domain, linker, and C-terminal are colored blue, orange, green, and magenta, respectively.

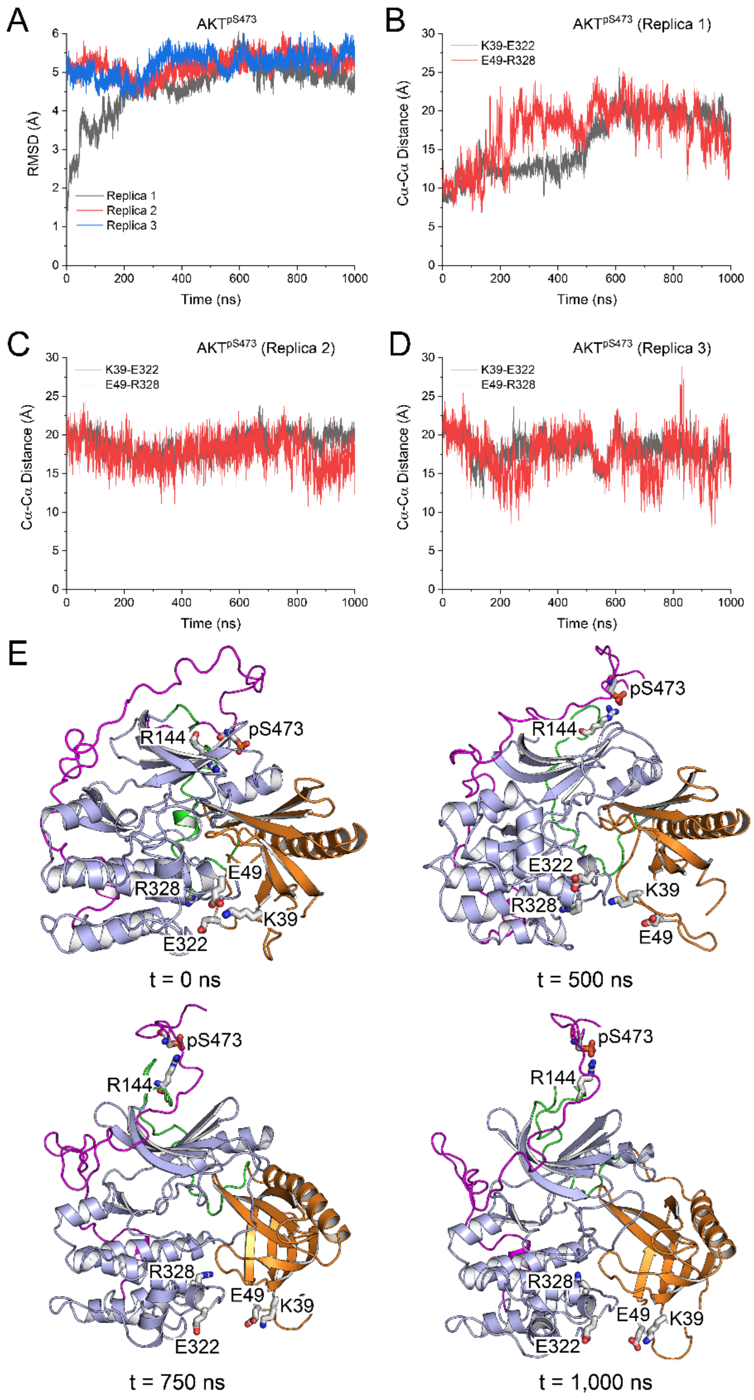


**Fig. S8.** (**A**) The root-mean-square deviation (RMSD) of Cα atoms of AKT$^{pS473}$ during the simulations. The linker and C-terminal region are excluded for structural alignment. (**B–D**) The changes in the distance of K39-E322 and E49-R328 in three replica simulations. K39 and E49 are in the variable loop 2 which undergoes substantial conformational changes. (**E**) Structural snapshots taken from the trajectory of replica 1 at different simulation time scales, showing the conformational dynamics of the variable loop 2. The kinase domain, PH domain, linker, and C-terminal are colored blue, orange, green, and magenta, respectively.

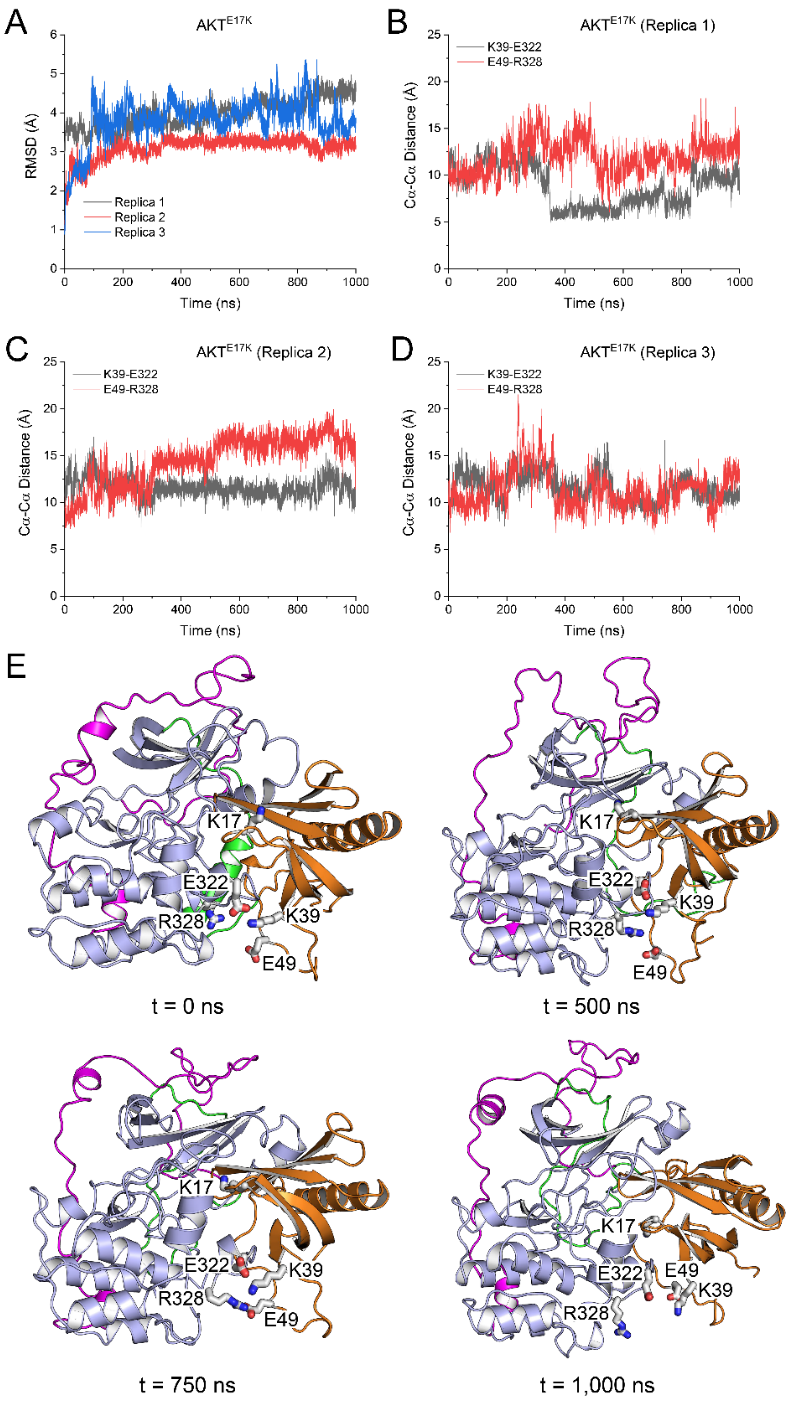


**Fig. S9.** (**A**) The root-mean-square deviation (RMSD) of Cα atoms of AKT$^{E17K}$ during the simulations. The linker and C-terminal region are excluded for structural alignment. (**B–D**) The changes in the distance of K39-E322 and E49-R328 in three replica simulations. K39 and E49 are in the variable loop 2 which undergoes substantial conformational changes. (**E**) Structural snapshots taken from the trajectory of replica 1 at different simulation time scales, showing the conformational dynamics of the variable loop 2. The kinase domain, PH domain, linker, and C-terminal are colored blue, orange, green, and magenta, respectively.

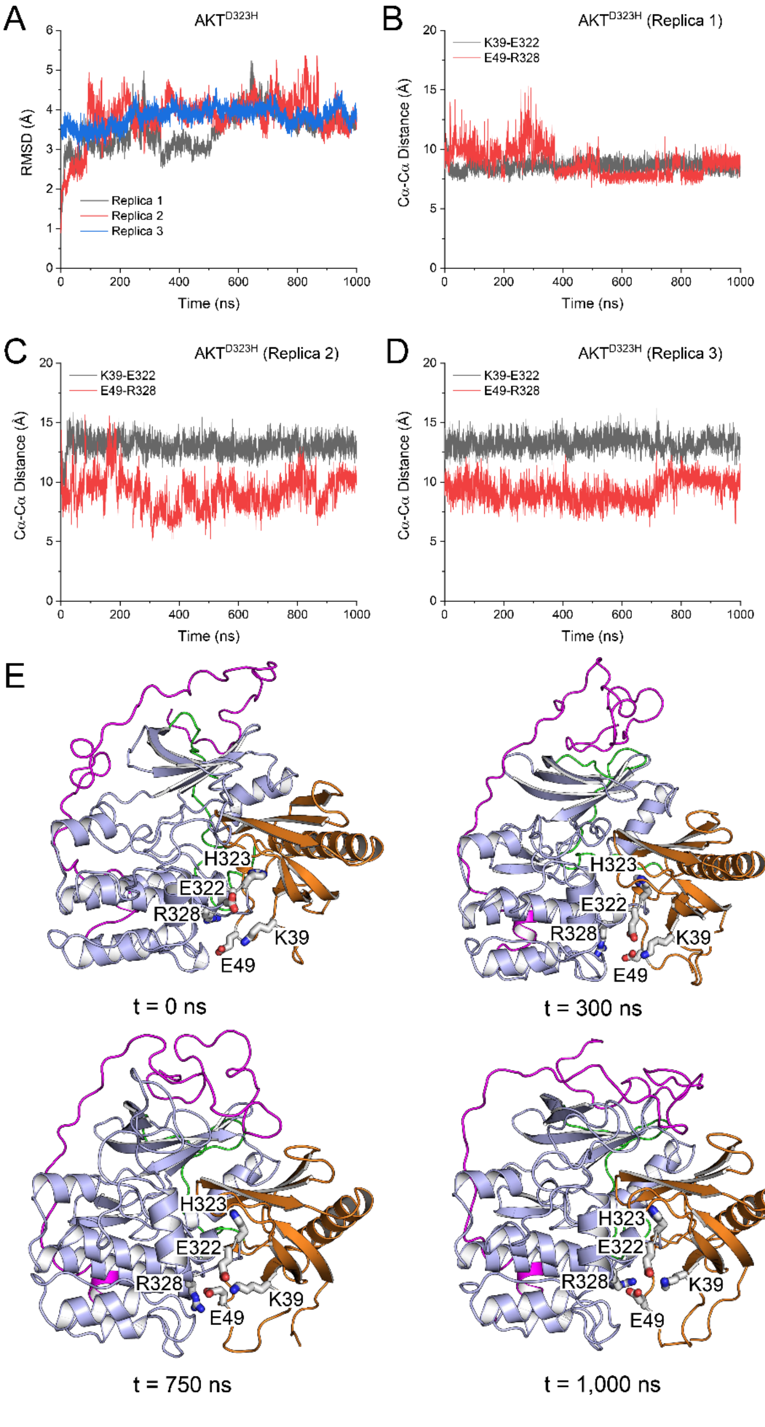


**Fig. S10.** (**A**) The root-mean-square deviation (RMSD) of Cα atoms of $AKT^{D323H}$ during the simulations. The linker and C-terminal region are excluded for structural alignment. (**B–D**) The changes in the distance of K39-E322 and E49-R328 in three replica simulations. K39 and E49 are in the variable loop 2 which undergoes substantial conformational changes. (**E**) Structural snapshots taken from the trajectory of replica 1 at different simulation time scales, showing the conformational dynamics of the variable loop 2. The kinase domain, PH domain, linker, and C-terminal are colored blue, orange, green, and magenta, respectively.

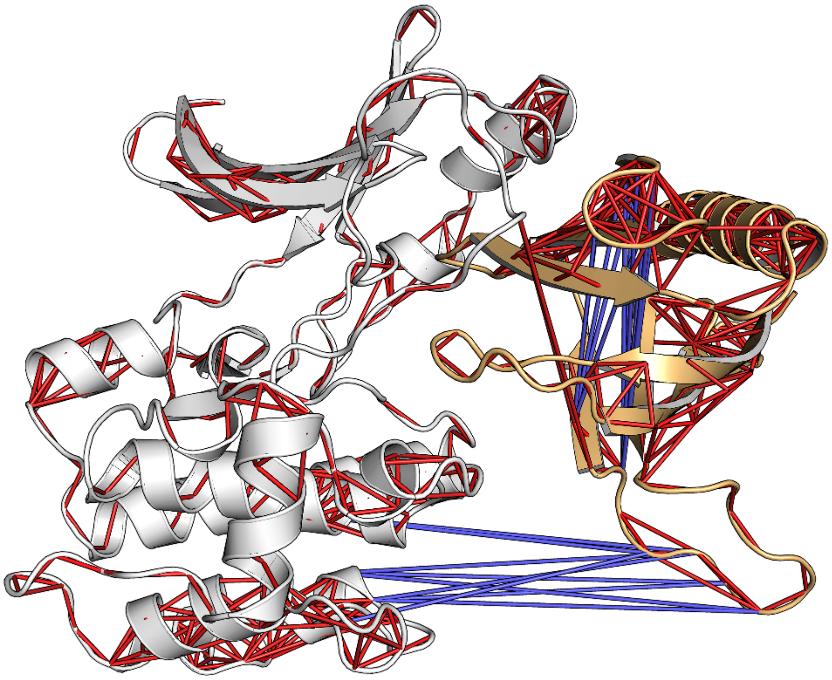

**Fig. S11. The most positively correlated motion (red) and negatively correlated motion (blue) in AKT$^{pS473}$.** The $C_{ij}$ of the most correlated motion (red) is in [0.8, 1.0], and the $C_{ij}$ of the most anticorrelated motion (blue) is in [-0.8, -0.6]. There are strong anticorrelated motions between the C-lobe of the kinase domain and the PH domain, as well as within the PH domain.

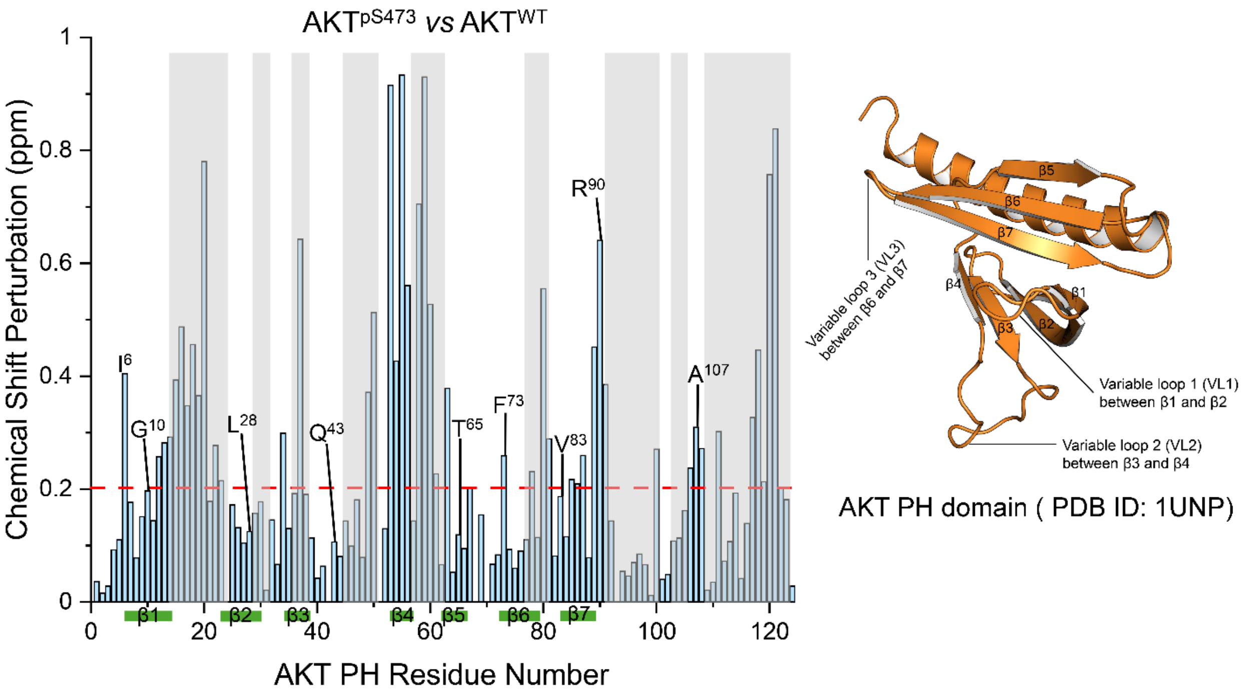


**Fig. S12. Chemical shift perturbations of AKT$^{pS473}$ with respect to AKT$^{WT}$.** The chemical shift of each residue of PH in AKT$^{WT}$ and AKT$^{pS473}$ was calculated using the SHIFTX2 program, which can accurately predict protein chemical shifts (*J Biomol NMR*, 2011, 50, 43). The combined chemical shift perturbation (CSP) for each PH residue was then calculated according to the equation $\Delta\delta = \sqrt{\Delta\delta_H^2 + (0.154 \times \Delta\delta_N)^2}$ where $\Delta\delta_H$ and $\Delta\delta_N$ are the changes in the chemical shift of proton and nitrogen on the backbone of an amino acid, respectively, and 0.154 is the scaling factor on the N nuclei that balances the changes in chemical shifts between the two nuclei (*Protein Sci*, 2021, 30, 2069). The average chemical shift for each PH residue was calculated based on the conformations extracted from three replicate simulations of AKT$^{WT}$ and AKT$^{pS473}$, respectively. Multiple residues in different regions that were assigned experimentally are labeled to help identify different β regions. The structure of the AKT PH domain is also shown, with the secondary structures labeled. The gray areas denote disordered regions between two β-strands. The red dashed line represents the standard deviation. The chemical shifts were calculated using SHIFTX2 program installed on NMRbox (*Biophys J.*, 2017, 112, 1529).

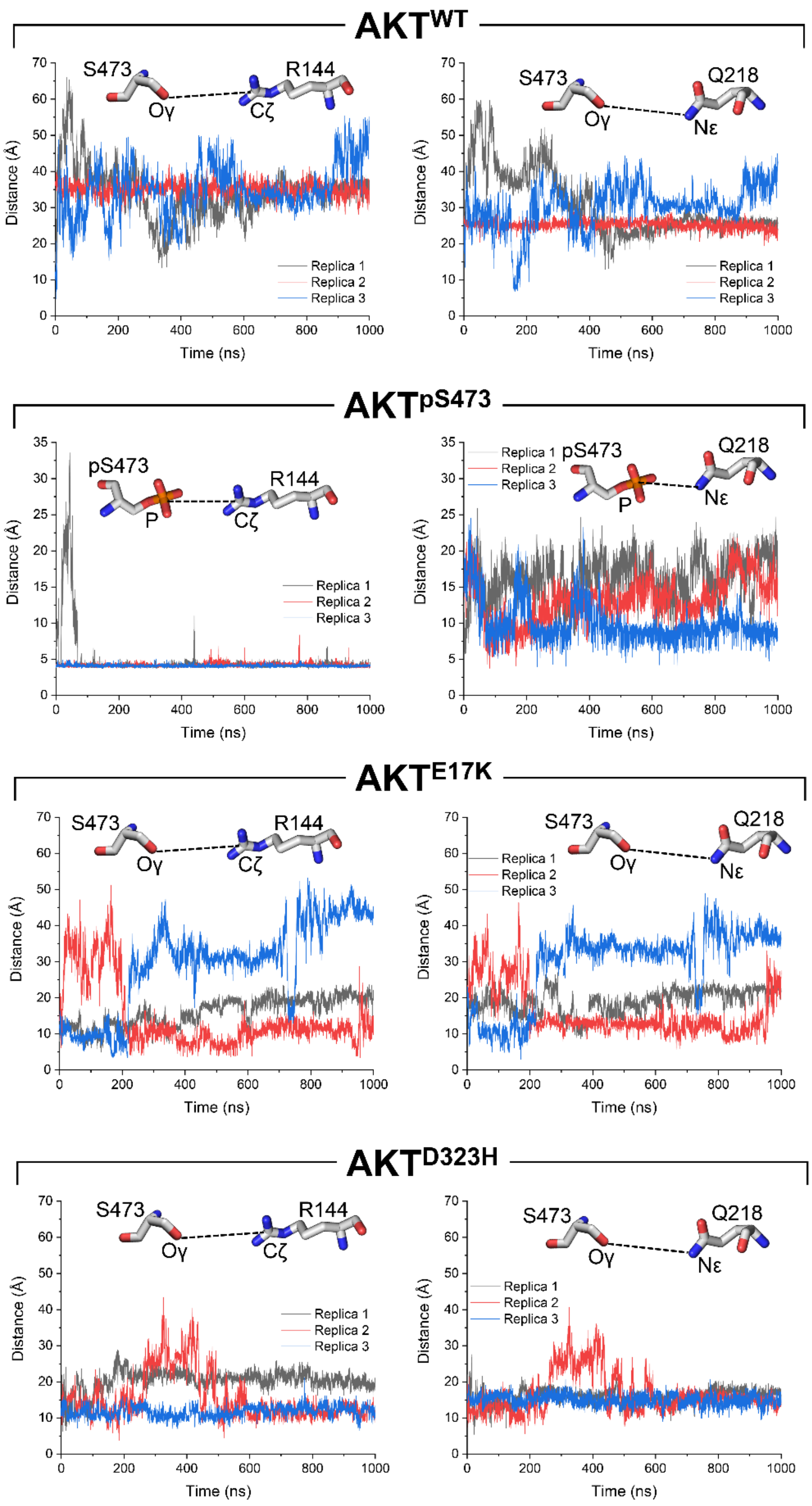


**Fig. S13. The change in the distance of S473/pS473-R144 and S473/pS473-Q218 with simulation time in different systems.** The atom pairs used to measure distance are shown.

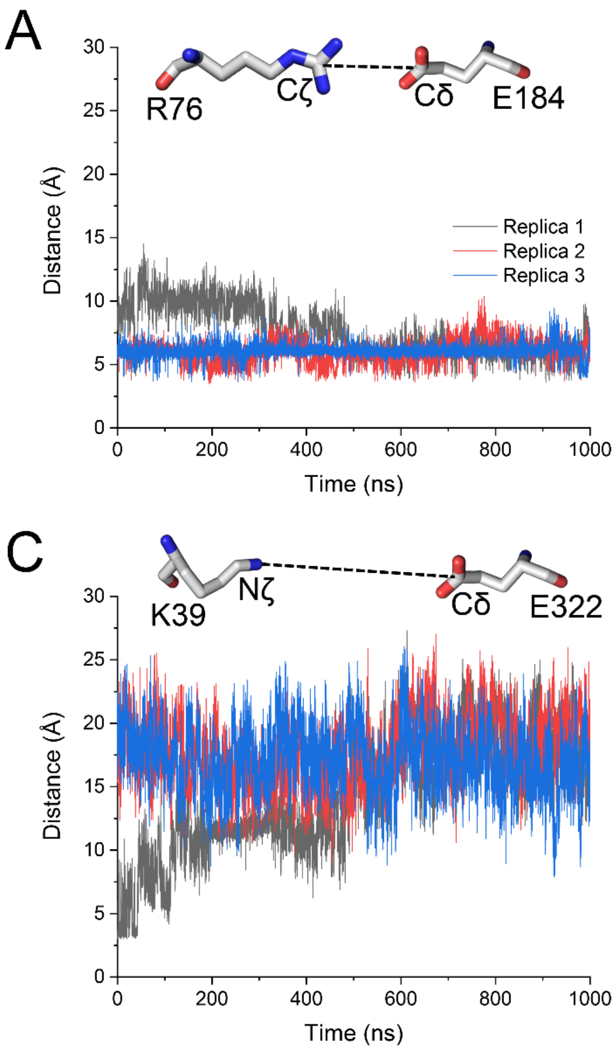


**Fig. S14. The change in the distance of R76-E184, K111-E191, and K39-E322 in the simulations of AKT$^{pS473}$.** The atom pairs used to measure distance are shown.

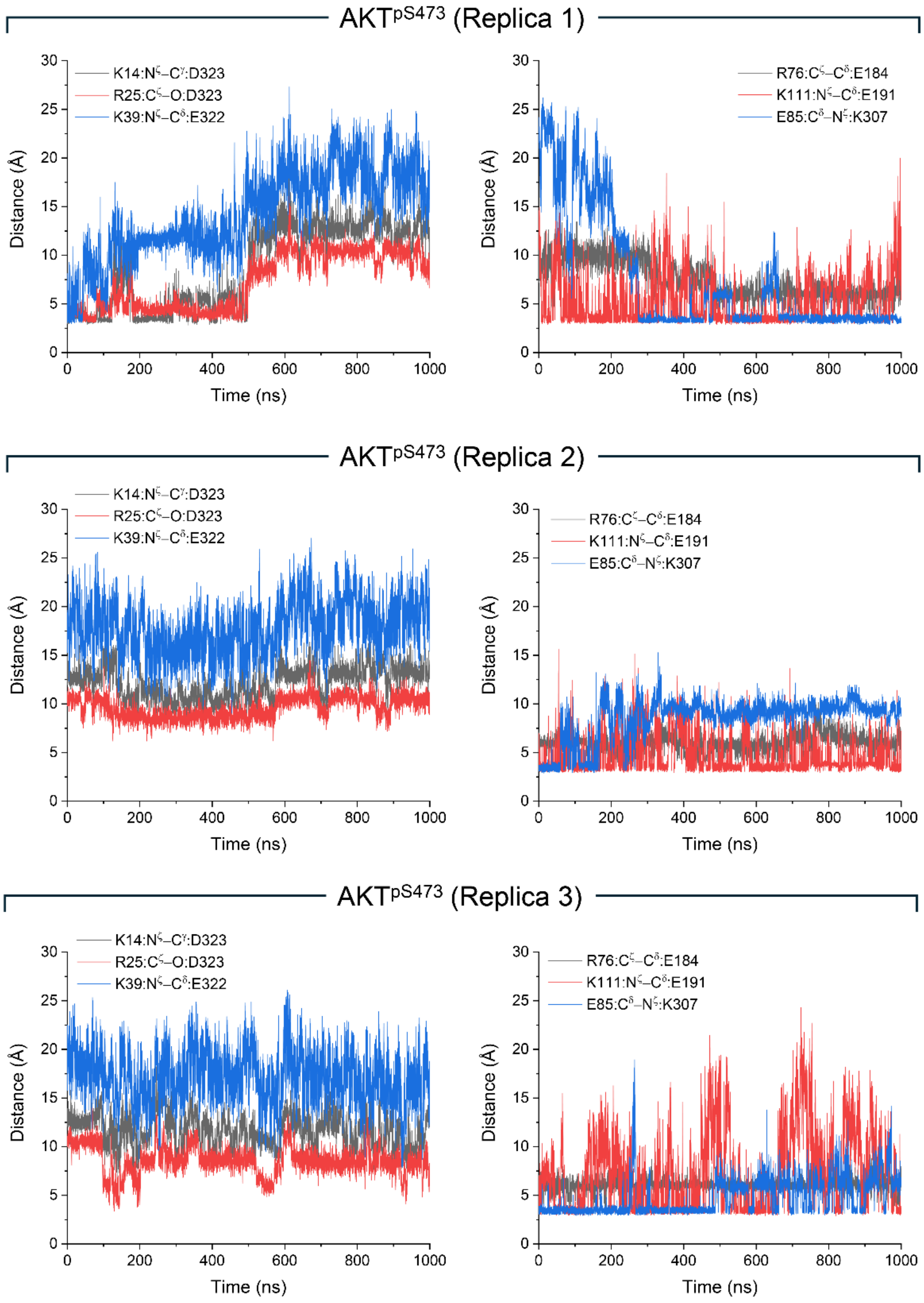


**Fig. S15. The changes in the salt bridges at the autoinhibitory interface of AKT$^{pS437}$.** In all replicate simulations, the salt bridges K14–D323, R25–D323, and K39–E322 were lost after 500 ns. On the other hand, the salt bridges R76–E184, K111–E191, and E85–K307 were dynamically established throughout simulations. No coordinated changes in the occupancy of the two sets of salt-bridge interactions were observed. The interaction between pS473 and R144 allosterically triggers the formation of the new salt bridges.

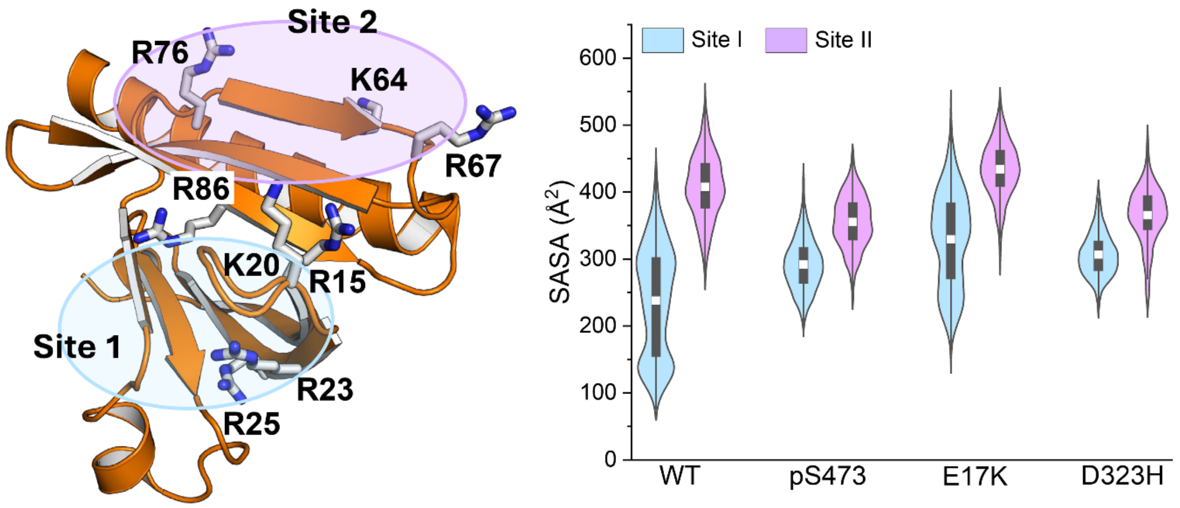


**Fig. S16. Distinct SASAs of the two lipid binding sites in the PH domain of AKT.** The canonical lipid binding site (Site 1) includes R15, K20, R23, and R25; and the second lipid binding site (Site 2) includes K64, R67, R76, and R86. The solvent accessible surface area (SASA) was calculated for those charged residues in each binding site. Unlike **Fig. 7**, the present plot shows the distributions of the SASAs of all trajectories of each system. For each binding site, two sample *t* tests were performed for the paired dataset between WT and non-WT (pS473, E17K, and D323H), and a value of $p < 0.001$ was obtained consistently.

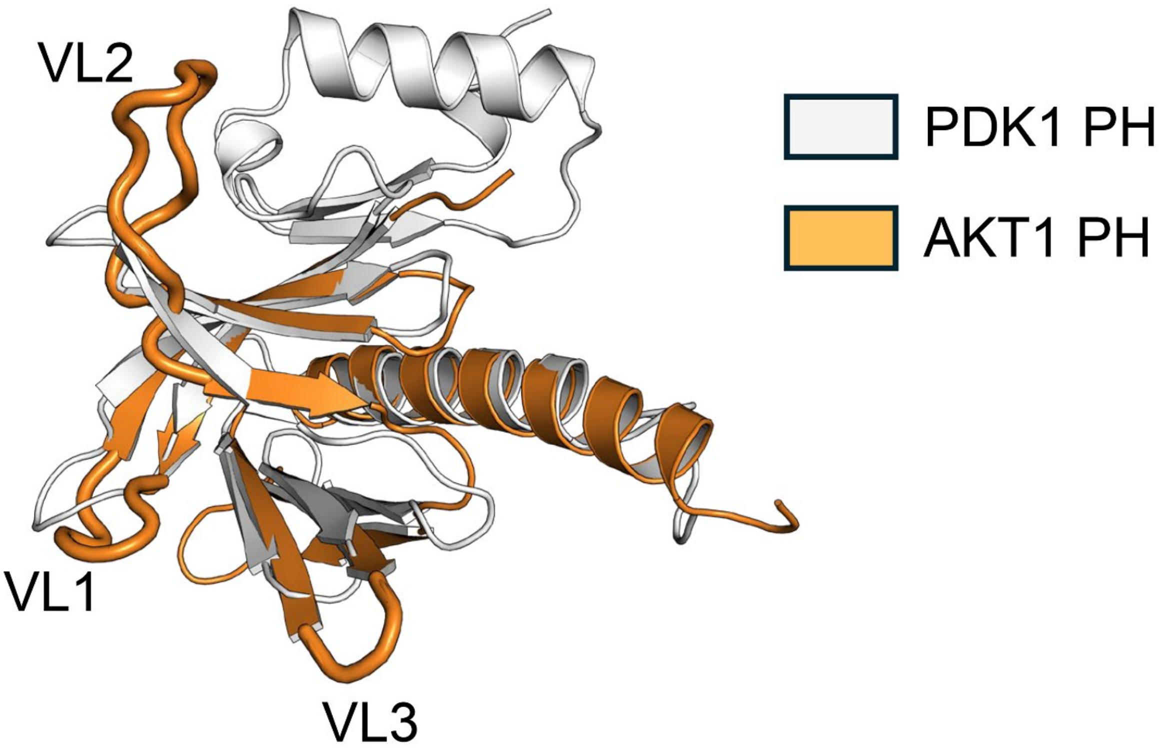


**Fig. S17. Structural superposition of the AKT PH domain (PDB ID: 1UNQ) with PDK1 PH domain (PDB ID: 1W1H).** The three variable loops (VL1, VL2, and VL3) are labeled and appear to be more flexible in the AKT PH domain.

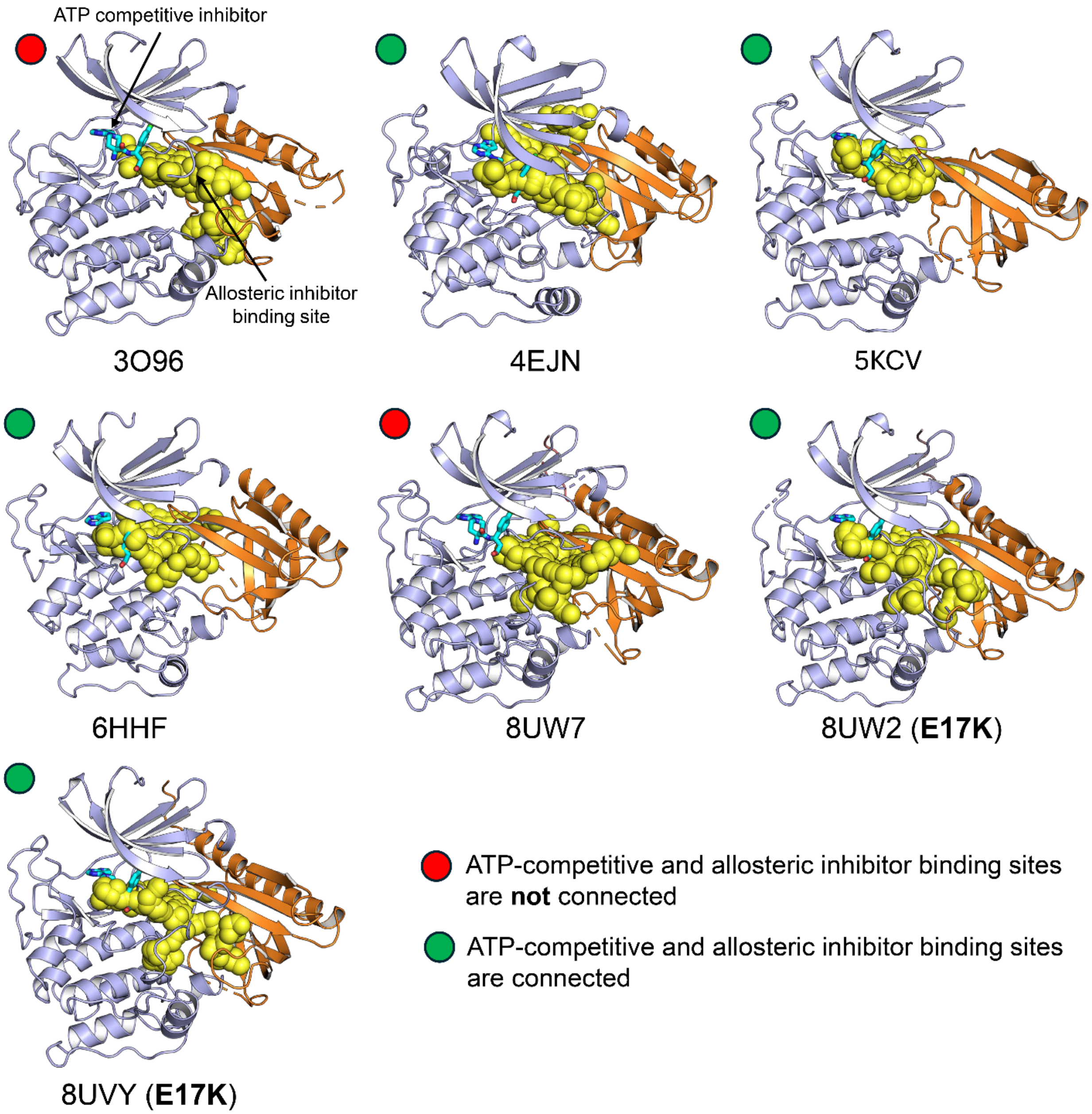


**Fig. S18. The binding pockets in the crystal structures of autoinhibited AKT.** The binding site for the ATP-competitive inhibitor (cyan stick) is obtained by superimposition of the crystal structure of AKT kinase domain in complex with capivasertib (Truqap) (PDB ID: 4GV1) with individual crystal structure of autoinhibited AKT. The yellow spheres display the continuously accessible cavities detected by the program Fpocket (*BMC Bioinformatics* 2009, 10, 168). In two crystal structures (PDBs: 3O96 and 8UW7), the ATP-competitive and allosteric inhibitor binding sites are not connected, but the two binding sites seem connected in the other crystal structures of autoinhibited AKT.

**Table S1.** The pair residues that contribute to the electrostatic interactions shown in Fig. S2. VL1, Vl2, and VL3 denote the variable loop 1, 2, and 3 in the PH domain, respectively.

| PDB | **VL1 (15–22)** | **VL2 (38–53)** | **VL3 (79–82)** |
|---|---|---|---|
| 3O96 | K14-D323; R23-E322; R25-D323; | K39-D325 | |
| 4EJN | K14-D323; R23-E322; R25-D323; | | E85-K297 |
| 5KCV | K14-D323; R23-E322; R25-D323 | | |
| 6S9W | K14-D323; E17-R273; K20-E298; R23-E322; R25-D323 | K39-E322; E49-R328 | |
| 6HHF | E17-R273; R23-D323; R25-E322 | | K76-E184 |
| 7APJ | | | |
| 8UW7 | K14-D323; E17-R273; R23-E322; R25-D323 | K39-D325 | |
| $8UW2^{E17K}$ | K14-D323; K17-E298; R23-D323; R25-D323 | K39-E322 | |
| $8UVY^{E17K}$ | K14-D323; R23-E322; R25-D323 | K39-D325 | |
